# Design of a bioactive small molecule that targets r(AUUCU) repeats in spinocerebellar ataxia 10

**DOI:** 10.1038/ncomms11647

**Published:** 2016-06-01

**Authors:** Wang-Yong Yang, Rui Gao, Mark Southern, Partha S. Sarkar, Matthew D. Disney

**Affiliations:** 1 Departments of Chemistry and Neuroscience, The Scripps Research Institute, Scripps Florida, Jupiter, Florida 33458, USA; 2 Mitchell Center for Neurodegenerative Disorders, Department of Neurology, Neuroscience and Cell Biology, University of Texas Medical Branch, Galveston, Texas 77555, USA; 3 Informatics Core, The Scripps Research Institute, Scripps Florida, Jupiter, Florida 33458, USA

## Abstract

RNA is an important target for chemical probes of function and lead therapeutics; however, it is difficult to target with small molecules. One approach to tackle this problem is to identify compounds that target RNA structures and utilize them to multivalently target RNA. Here we show that small molecules can be identified to selectively bind RNA base pairs by probing a library of RNA-focused small molecules. A small molecule that selectively binds AU base pairs informed design of a dimeric compound (**2AU-2**) that targets the pathogenic RNA, expanded r(AUUCU) repeats, that causes spinocerebellar ataxia type 10 (SCA10) in patient-derived cells. Indeed, **2AU-2** (50 nM) ameliorates various aspects of SCA10 pathology including improvement of mitochondrial dysfunction, reduced activation of caspase 3, and reduction of nuclear foci. These studies provide a first-in-class chemical probe to study SCA10 RNA toxicity and potentially define broadly applicable compounds targeting RNA AU base pairs in cells.

## Abstract (web summary)

Expanded RNA repeats in non-coding region of a gene represent a hallmark of several diseases. Here, the authors identify two small molecules that selectively bind AU repeats and use them to design a compound that targets the pathogenic RNA associated with spinocerebellar ataxia type 10.

## Graphical abstract

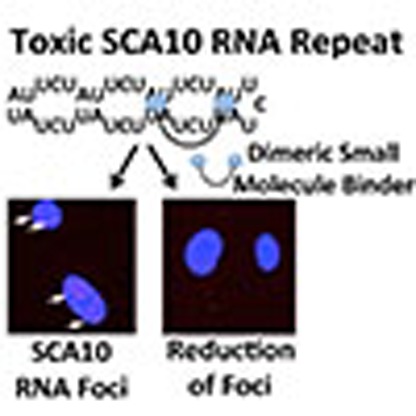

RNA has diverse cellular functions. For example, messenger RNAs (mRNAs) encode protein, microRNAs regulate the lifetime of mRNAs, and the ribosome translates mRNAs into proteins^1^,^2^. In bacteria, riboswitches control the production of proteins by binding to small molecule metabolites^3^,^4^. In fact, many non-coding RNAs have been found to play significant roles in cellular biology, and these discoveries expand even further the known functions of RNA^5^.

Because of the important cellular functions of RNA under normal conditions, it is not surprising that mutations in RNA can cause disease. Single-nucleotide polymorphisms can give rise to cryptic alternative pre-mRNA splicing sites, leading to production of aberrant, defective proteins, as is the case with β-thalassaemia^6^,^7^. Expanded RNA repeats can also contribute to disease and can be present in 5′ and 3′ untranslated regions (UTRs; fragile X-associated tremor ataxia syndrome (FXTAS)^8^ and myotonic dystrophy type 1 (DM1)^9^), introns (spinocerebellar ataxia type 10 (SCA10)^10^ and myotonic dystrophy type 2 (DM2)^11^) or coding regions (Huntington's disease (HD)^12^). Small molecules that target these RNA and inhibit its dysfunction are thus highly desirable.

The bacterial ribosome is the most widely exploited RNA target^13^,^14^. Ribosomes and ribosomal RNA (rRNA) are privileged targets as (i) ribosomes play essential roles in cellular homeostasis and modulation of ribosome activity can have drastic cellular consequences, and (ii) rRNA comprises about 80–90% of the total RNA content of a cell^15^. Riboswitches are also emerging and established targets of RNA-directed small molecules. Compounds identified to bind to and modulate riboswitches generally mimic the structure of the RNA's natural metabolite^3^,^16^, akin to substrate mimicry to design enzyme inhibitors^17^. Most RNA targets to which a small molecule binder is desired, however, are of low abundance and have no natural metabolite to inform drug design.

To aid RNA-targeting endeavours, our group has developed two-dimensional combinatorial screening (2DCS) to identify optimal (high affinity and selective) RNA motif–small molecule interactions. These interactions are deposited into a database and can be used to design small molecules to target RNAs by comparing motifs in the desired target to the database. This approach has been used to design small molecules targeting the RNAs that cause DM^18^,^19^,^20^, FXTAS^21^ and HD^22^. All of the interactions that are presently in the RNA motif–small molecule database are between small molecules and RNA loops such as hairpins, bulges and internal loops. Herein, we report small molecules that bind selectively to RNA base pairs. Among a variety of compounds tested, small molecules with benzamidine moieties were identified to bind selectively to AU base pairs. These data were leveraged to design the first bioactive small molecule to target the expanded r(AUUCU) repeat that causes SCA10, an incurable neuromuscular disorder. The compound targets the central AU base pairs in r(AUUCU)^exp^ and its dimeric compound (**2AU-2**) displaces sequestered proteins and improves defects in patient-derived cells. The observation that base pair-binding modules can provide bioactive compounds suggests that many other RNAs can be exploited as targets of small molecules.

## Results and Discussion

### Binding of RNA-focused small molecules to RNA base pairs

**Figure 1:**
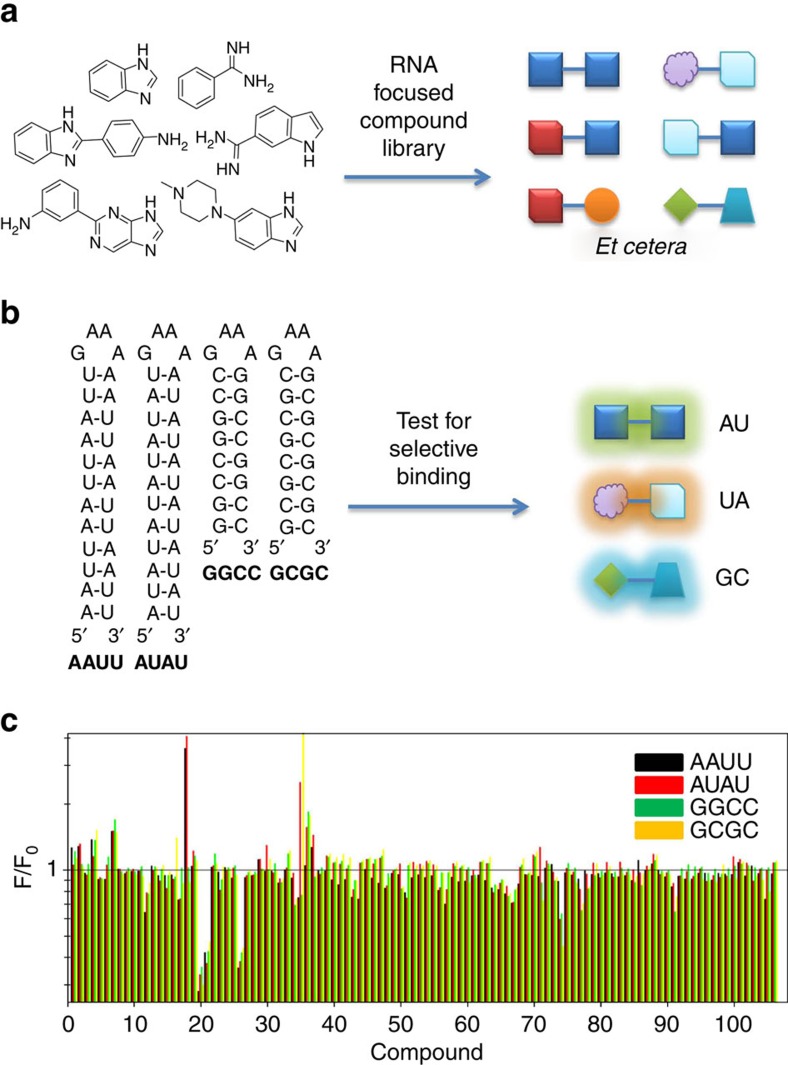
Overall scheme to identify small molecules that bind to RNA base pairs. (**a**) A small molecule library was collected in which the small molecules have chemotypes present in compounds known to bind RNA. (**b**) The RNA-focused small molecule library was tested for binding to four different RNAs that have different orientations and identities of RNA base pairs. (**c**) Compounds were tested in a fluorescence emission assay for selectively binding to RNAs with different paired elements. Compounds that were selective were further analysed.

By using chemical similarity searching, small molecules with features that should pre-dispose them for binding RNA^23^,^24^ were collected from both the National Cancer Institute's and The Scripps Research Institute's chemical libraries, including benzimidazole, benzamidine, aniline moieties. Compounds were further restrained to be fluorescent to allow for easy screening of binding events, affording 104 small molecules (Fig. 1a and Supplementary Table 1).

Compounds were screened for binding to different base pairs using four model constructs with stretches of AU and GC base pairs embedded in the stem of a common hairpin loop (Fig. 1b). The four RNAs display AU or GC pairs with different nearest neighbours. AUAU and AAUU RNAs have 5′AU/3′UA or 5′AAUU/3′UUAA stretches, respectively, while GCGC and GGCC have 5′GC/3′CG or 5′GGCC/3′CCGG stretches, respectively. Due to significant differences in the thermodynamic stability of the hairpins, RNAs with AU pairs had 12 base pairs in the stem while RNAs with GC pairs had 8 (ref. 25). The Δ*G*°_37_ for AUAU and AAUU is −8.5 and −6.7 kcal mol^−1^, respectively, while for GCGC and GGCC it is −17.5 and −19.1 kcal mol^−1^, respectively. If only eight AU base pairs were present, then the free energy drops considerably to −3.7 and −2.5 kcal mol^−1^, respectively.

In initial compound screens, each small molecule was incubated with the four RNAs and the change in emission was measured (Fig. 1c). Changes in emission were not only analysed for statistical significance for binding to these RNAs in general, but also for binding to the different RNA structures. For this compound collection, 29% of the compounds exhibited a change in emission upon binding to any of the RNAs used in this study, and 18% of the compounds showed preferential binding to AU or GC pair-containing duplexes (Supplementary Table 2).

**Figure 2:**
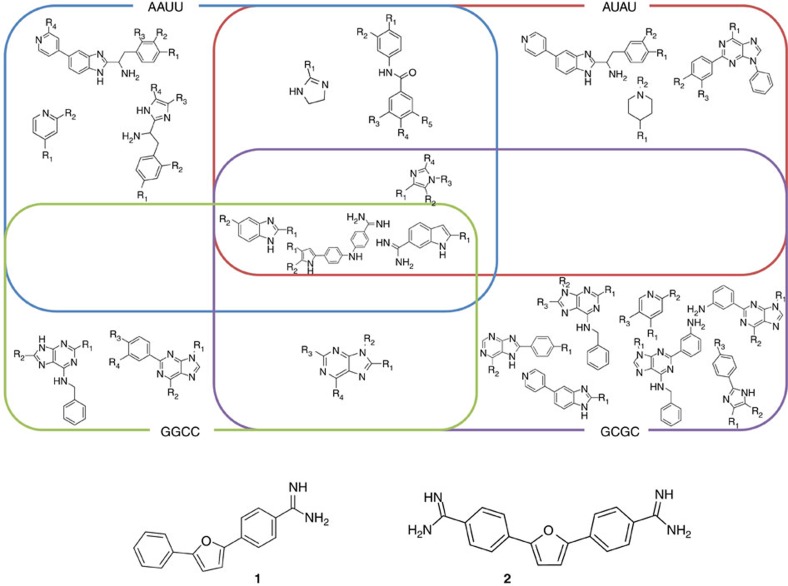
Analysis of compounds identified to bind RNA base pairs. Top, Venn diagram of substructures in compounds that were found to bind to RNA from the fluorescence screening assay showed in Fig. 1c. Data were compiled by using compounds that had a *P* value of <0.001 for binding to the RNA hairpins. Bottom, structures of compounds **1** and **2** that were the most avid for binding to AUAU and AAUU RNA hairpins.

On the basis of the substructures within compounds that bind, a Venn diagram was constructed to correlate chemotypes and binding to AU and GC base pairs (Fig. 2). For example, various functionalized purines bound to RNAs with GC base pairs. Previously, other compounds that bind RNA base pairs have been identified. For example, the Beal group has used threading intercalators with acridine or other aromatic functionalities to target RNA bulges with nearby GC pairs^26^. Thus, it appears that acridine, and related compounds, can provide a GC base-pair-binding module. The group of Tor has shown that ethidium bromide is a 20-fold selective binder to poly r(A)-poly r(U) over poly r(G)–poly r(C)^27^.

Each small molecule from the initial screen was tested for saturable binding when incubated with serially diluted RNA. The benzamidine compounds **1** and **2** provided the most robust and saturable change in emission and were found to bind selectively to AU base pairs (Supplementary Figs 1, 2 and 3). Due to the binding of these compounds to AU base pairs and their saturable emission properties, these compounds were selected for further study. This assay, by nature of using emission to study binding, could generate false negatives; however, it allowed us to further characterize positive hits and produce a first-in-class bioactive ligand that targets r(AUUCU), *vide infra*.

### Compounds 1 and 2 bind selectively to AU base pairs

**Figure 3:**
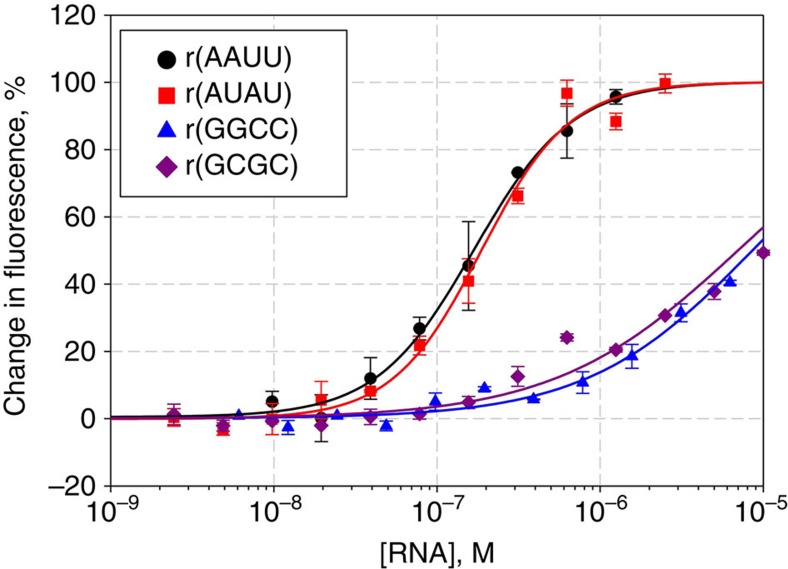
Binding of compound 2 to four different RNAs Fluorescence binding assays of compound 2 to RNAs with different base pairs show that 2 binds AU base pairs selectively over GC base pairs by >40-fold.

The selectivities of compounds **1** and **2** were further assessed by measuring EC_50_s for all four RNAs. The EC_50_s for **1** for binding to AAUU, AUAU, GGCC and GCGC are 170, 45, 390 and 240 nM, respectively, while the EC_50_'s for **2** are 170, 190, 8,550 and 6,950 nM, respectively (Table 1). On the basis of these data, **2** is much more selective for AU base pairs than **1,** with an AU pair selectivity of ∼40-fold (Fig. 3). Measurement of the *K*_d_'s of **2** to AUAU and AAUU give values of 210 and 320 nM, respectively, and stoichiometries of 2.9 and 3.7, respectively (Supplementary Fig. 4). These data suggests that **2** is a high affinity binder to stretches of AU base pairs and that the compound interacts with between 3 and 4 base pairs in each of the model hairpins. Previous studies have suggested that the aminoglycoside tobramycin recognizes model poly(rI)·poly(rC) RNA duplexes and that the compound interacts with approximately 4 bp, or a similar number of interacting sites that are proposed for **2** (ref. 28). Diphenylfuran amidine was previously reported to bind polyr(A)·polyr(U) duplex via intercalation, as determined from viscosity and circular dichroism studies^29^,^30^. These observations were further supported by docking studies in which the intercalation of **2** is stabilized through stacking interactions with the positively charged amidine residing in the major groove; minor groove binding could not be accommodated^29^,^30^. Further, the substituents in this class of compounds affect the thermal stability of polyr(A)·polyr(U), with imidazoline providing the largest enhancement^29^,^30^.

### Compounds 1 and 2 bind r(AUUCU) repeats that cause SCA10

**Figure 4:**
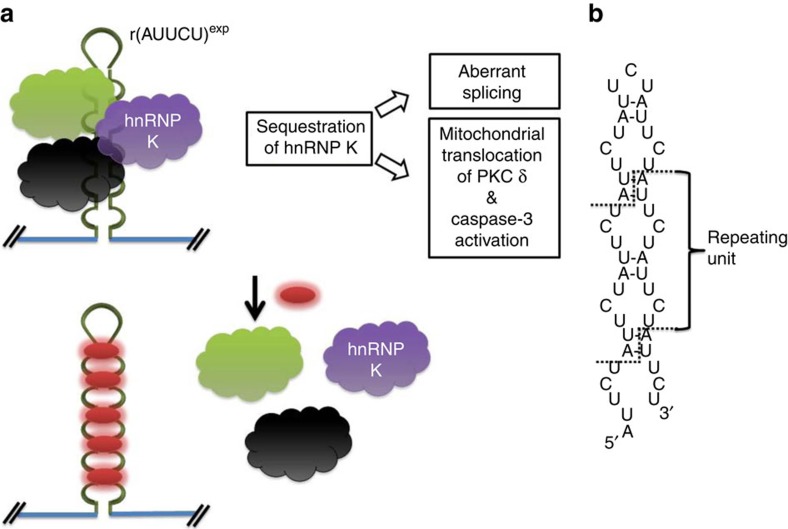
Schematic of the pathogenic mechanism of SCA10 and a therapeutic approach by targeting the secondary structure of r(AUUCU) repeats. (**a**) r(AUUCU)^exp^, located within intron 9 of ataxin 10 (*ATX10*) mRNA, sequesters proteins including hnRNP K. Sequestration of hnRNP K results in aberrant splicing of transcripts, mitochondrial translocation of PKCδ and caspase-3 activation, leading to apoptosis (top). A possible therapeutic approach is using small molecules that bind r(AUUCU) repeats and displace sequestered proteins (bottom). (**b**) secondary structure of r(AUUCU) repeats with periodically repeating 5′AU/3′UA base pair steps.

The ultimate goal of identifying small molecules that bind RNA motifs *in vitro* is to apply these identified interactions to target an RNA that causes disease. Fortuitously, the expanded repeating r(AUUCU) RNA (r(AUUCU)^exp^) that causes spinocerebellar ataxia type 10 (SCA10) contains stretches of AU base pairs^10^, in particular repeating 5′AU/3′UA base pair steps (Fig. 4b)^31^. SCA10 is an incurable neuromuscular disorder that is mainly found in Latin America^32^. It is a slowly progressive disease that results in poor balance followed by loss of control over upper limbs. Previous experiments have shown that the disease is due to an RNA gain-of-function in which r(AUUCU)^exp^, located within spliced intron 9 of the ataxin 10 (*ATX10*) mRNA, binds to and sequesters proteins involved in RNA biogenesis such as heterogeneous nuclear ribonucleoprotein K (hnRNP K; Fig. 4a)^33^. Sequestration of hnRNP K causes a host of cellular defects that include the formation of RNA nuclear foci, translocation of protein kinase C-δ (PKCδ) in mitochondria resulting in mitochondrial dysfunction, and activation of caspase 3 and subsequent apoptosis^33^. The binding of the repeats to RNA-binding proteins causes the transcript to be retained in nuclear foci in patient-derived cells and model cellular systems^33^.

**Figure 5:**
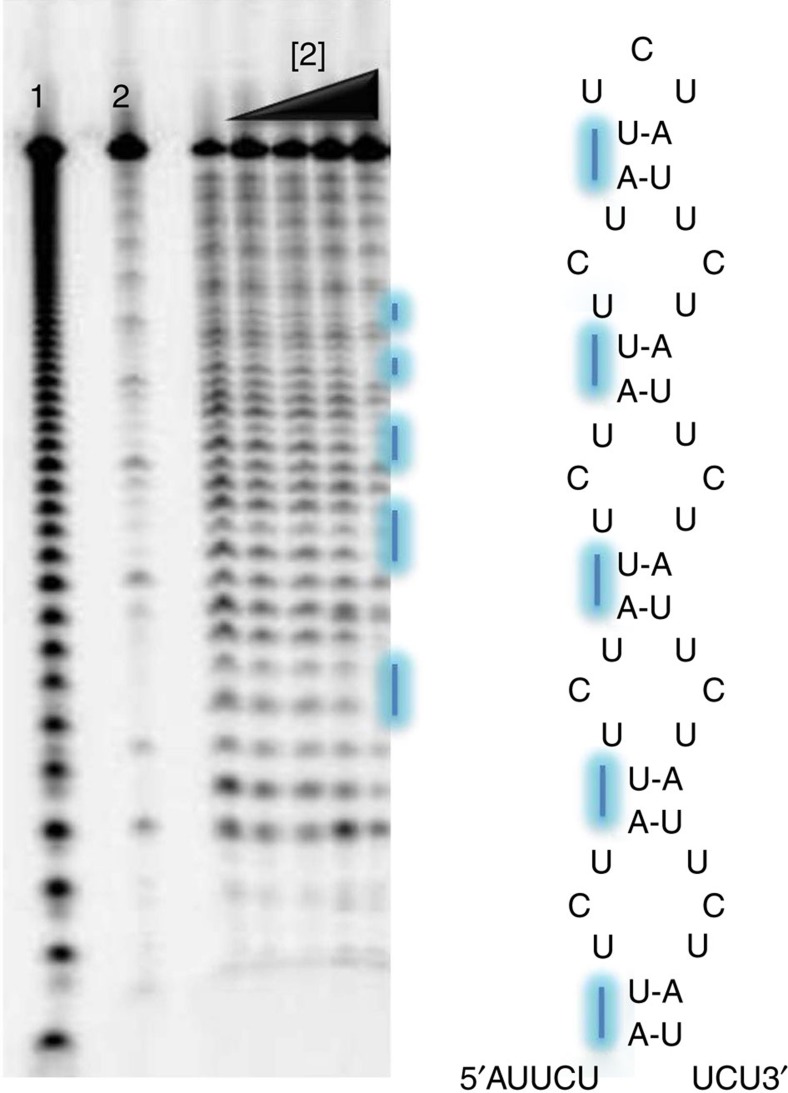
Compound 2 protects r(AUUCU)_11_ from cleavage by RNase V1. Left, representative gel image of the RNase protection assay. Lane 1, alkaline hydrolysis of r(AUUCU)_11_; lane 2, r(AUUCU)_11_ + No RNase V1; lanes 3–7, r(AUUCU)_11_ + RNase V1+**2** (0, 0.02, 0.2, 2 and 20 μM). Lines indicate sites of protection. Right, secondary structure of r(AUUCU)_11_. Protected nucleotides are indicated with blue lines.

We assessed the selectivities of compounds **1** and **2** for binding r(AUUCU) repeats over other disease-associated RNA repeats including r(CUG)_12_ (DM1)^9^, r(CGG)_12_ (FXTAS and FXS)^8^,^34^, and r(CCUG)_12_ (DM2)^11^. Compound **1** has a similar EC_50_ for all RNA repeats (36–87 nM), indicating that **1** binds to RNA internal loops as well as to AU base pairs with similar binding affinity (Supplementary Fig. 5). Compound **2** selectively recognizes r(AUUCU)_11_ with 5–15-fold selectivity over the other repeats (Table 2 and Supplementary Fig. 6). Compound **2** bound r(AUUCU)_11_ with a *K*_d_ of 300 nM and a stoichiometry of ca. 11:1 (**2**:r(AUUCU)_11_). Because r(AUUCU)_11_ contains 10 AU base pairs, it indicates that **2** is binding to each AU base pair in the target. Indeed, a nuclease protection assay revealed that **2** protects each AU base pair in r(AUUCU)_11_ from cleavage (Fig. 5). Interestingly, calculated Hill coefficients indicate that **2** binds to r(AUUCU)_11_ with positive cooperativity (*n*=1.7; Table 2), suggesting that **2** could be an ideal module to design a polyvalent compound to target multiple adjacent sites in r(AUUCU)^exp^ simultaneously.

### An AU base pair-binding module to target r(AUUCU)^exp^

**Figure 6:**
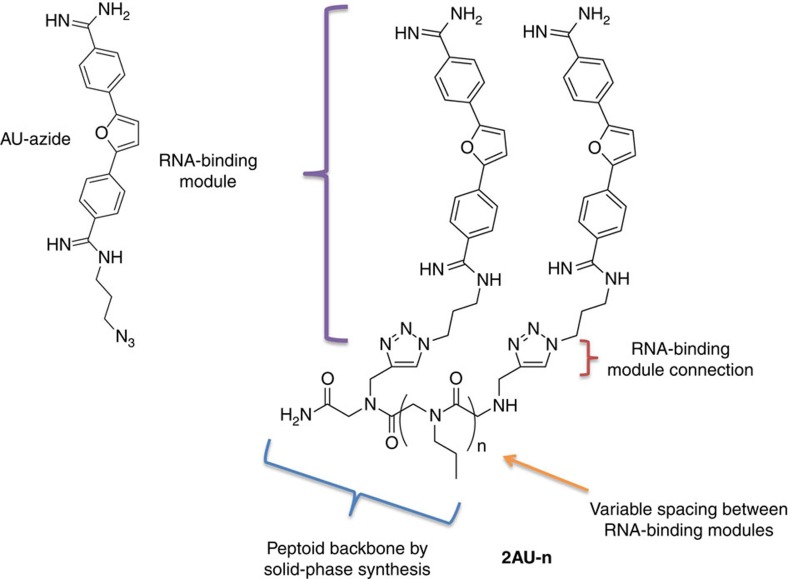
Design of dimeric compounds to target r(AUUCU)^exp^. The RNA-binding module, **AU-Azide** (left) was assembled onto various peptoid backbones to afford dimeric compounds (right). The **AU-azides** were separated by different distances by varying the number of spacing modules (n) to generate a series of compounds named **2AU-n**, where **2AU** denotes two RNA-binding modules and n denotes the number of spacing modules.

Previously, our group has developed modularly assembled small molecules that target various repeating RNAs^18^,^19^,^35^,^36^,^37^,^38^. In this approach, an RNA-binding module or modules that recognize different motifs in an RNA target are displayed on a single molecule. These polyvalent compounds allow for simultaneous recognition of multiple motifs in an RNA target, thereby increasing affinity and selectivity relative to monomeric binders (Fig. 6).

To enable this approach for **2** and r(AUUCU)^exp^, a derivative of **2**, **AU-azide** (Fig. 6), was synthesized to install an orthogonally reactive group. The azide is used for conjugation onto peptoid polyvalent scaffolds that display alkynes via a Cu-catalysed Huisgen cycloaddition reaction. Dibenzimidate (**3**) was synthesized from the furan via two reaction steps as reported previously^39^,^40^, followed by two amidation reactions to obtain **AU-azide** (Supplementary Fig. 19; see Supplementary Information for chemical synthesis details and compound characterization). After obtaining the desired compound, it was tested for selective recognition of AU over GC base pairs (Table 1 and Supplementary Fig. 7). Interestingly, **AU-azide** has enhanced selectivity of AU base pair than **2**, >45-fold selective for AU over GC base pairs (Table 1). The selectivity of **AU-azide** for r(AUUCU)_11_ over other RNA repeats compared with **2** was also improved (Table 2 and Supplementary Fig. 8).

### Development of dimeric compounds to target r(AUUCU)^exp^

We next synthesized a small library of **AU** dimers (Fig. 6) by using a previously published approach^35^,^36^,^37^. A peptoid backbone was used as a polyvalent scaffold that contains two propargylamine submonomers separated by different distances afforded by varying the number of propylamine spacers inserted into the backbone. The **AU-azides** (RNA-binding modules) were then conjugated by using a Cu-catalysed click reaction (Supplementary Fig. 21). The nomenclature used for these compounds is **2AU-n** where **AU** indicates the RNA-binding module; the number before **AU** indicates valency; and the number after the dash indicates the number of propylamine spacing modules between **AU** RNA-binding modules.

The library of dimeric compounds was then tested for binding to r(AUUCU)_11_ by using a filtre-binding assay (Supplementary Fig. 10). After incubation of radioactively labelled r(AUUCU)_11_ with dimeric compound, the compound-bound RNA was separated from free RNA by using a Dot-Blot apparatus. These studies showed that **2AU-2** bound to the RNA to the greatest extent, 2–20-fold greater than the other dimers. Additional filtre-binding assays were completed with 35-fold excess transfer RNA (tRNA) to gain insight into selectivity. Only a modest decrease (∼20%) of **2AU-2** binding was observed, suggesting the compound is selective.

To further confirm the results of the filtre binding assays, we developed an assay that evaluates the potency of small molecules for inhibiting protein loading onto r(AUUCU)_11_. It is known that DiGeorge syndrome critical region 8 (DGCR8), a protein that is involved in microRNA biogenesis, binds a wide variety of RNAs^41^ and thus could be of potential use as the protein component in this assay. To establish that DGCR8Δ binds r(AUUCU)_11_, a gel mobility shift assay was completed, affording a dissociation constant of 1.6 μM for the RNA–protein interaction (Supplementary Fig. 11). Such affinity is similar to those found between other RNA repeats and proteins, including r(CUG)^exp^-MBNL1 (muscleblind-like 1), r(CAG)^exp^-MBNL1, and r(CGG)^exp^-DGCR8 (refs 20, 21, 22).

**Figure 7:**
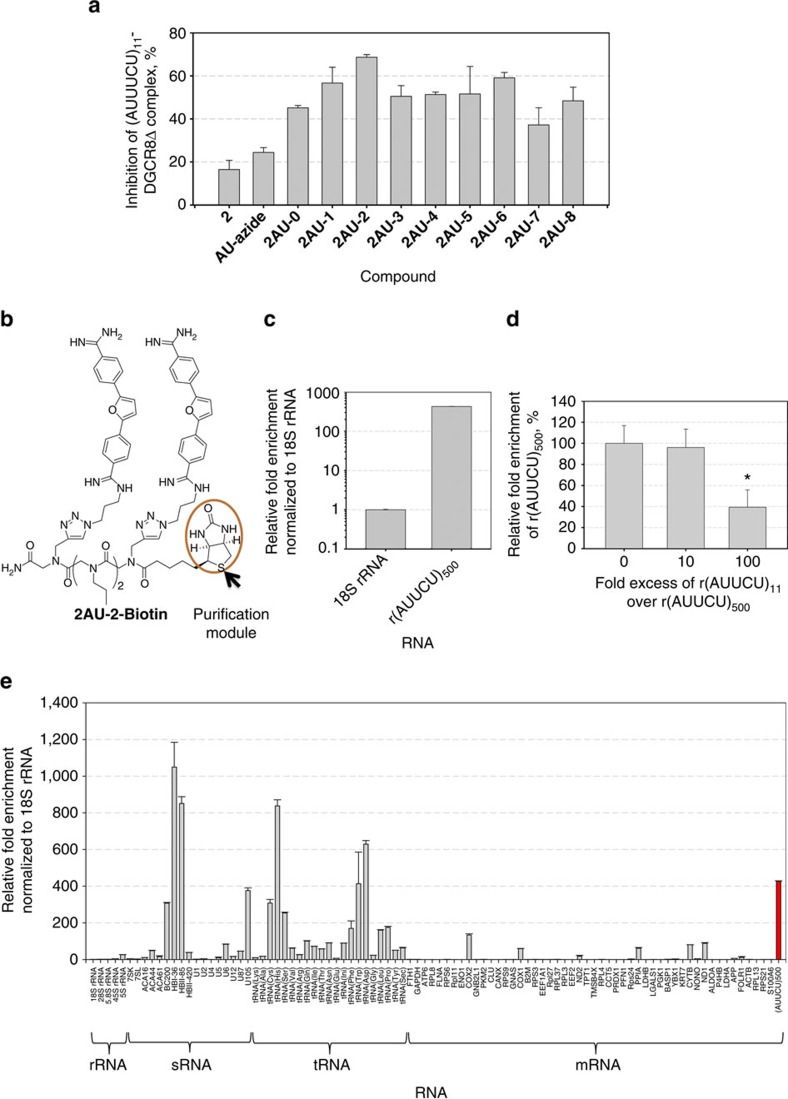
2AU-2 binds r(AUUCU) repeats *in vitro* and *in vivo* and inhibits protein binding. (**a**) quantification of inhibition of the r(AUUCU)_11_-DGCR8Δ complex by monomeric and dimeric compounds (1.5 μM). (**b**) structure of **2AU-2-Biotin**. (**c**) relative fold enrichment of r(AUUCU)_500_ pulled down by **2AU-2-Biotin** from total cellular RNAs. (**d**) **2AU-2-Biotin** selectively binds r(AUUCU)_500_ over r(AUUCU)_11_ as determined by a competition experiment. r(AUUCU)_500_ levels were normalized to 18S rRNA. (**e**) Profiling of 93 highly abundant RNAs to determine whether they bind **2AU-2-Biotin** in the context of total cellular RNA.

Time-resolved fluorescence resonance energy transfer (TR-FRET) assays have been developed to screen for inhibitors of each RNA–protein complexes mentioned above^20^,^21^,^22^. Thus, we developed a TR-FRET assay for r(AUUCU)_11_ and DGCR8Δ analogously. Screening results showed that dimers are better inhibitors than the monomer and that **2AU-2** is the most potent inhibitor amongst them (Fig. 7a). At 1.5 μM concentration, **AU-azide** only inhibits ∼25% of r(AUUCU)_11_-DGCR8Δ complex formation while **2AU-2** inhibits ∼70%. Further, the IC_50_ of **2AU-2** was 2.7-fold less than that of **AU-azide** (Supplementary Fig. 12). Thus, both the TR-FRET and filtre-binding assays establish that **2AU-2** is the most potent binder of r(AUUCU)_11_ and that there is considerable enhancement for the dimer relative to the monomer.

Consistent with results from filtre binding and TR-FRET assays, selective binding of **2AU-2** to r(AUUCU)_11_ over other RNA repeats was observed (Table 2 and Supplementary Fig. 9). The stoichiometry and the *K*_d_ of dimer **2AU-2** to r(AUUCU)_11_ was measured. The number of **2AU-2** per r(AUUCU)_11_ was approximately half that of **AU-azide** (*n*=7 and *n*=13, respectively), suggesting that each RNA-binding module interacts with each AU pair. The affinity of **2AU-2** for r(AUUCU)_11_ is twofold greater than the monomer and **AU-azide** and **2AU-2** maintained positively cooperative binding to r(AUUCU)_11_. Similar increases in affinity when increasing valency from *n*=*1* to *n*=*2* have been observed with other repeats^19^,^35^. Furthermore, we studied the binding of **2AU-2** and r(AUUCU)_11_ under molecular crowding conditions that mimic a cellular environment by adding 20% (w/v) PEG 8000. A ninefold enhancement in binding was observed (EC_50_ of 16±4 nM) relative to non-molecular crowding conditions (EC_50_ of 146±8 nM; Supplementary Fig. 13).

The binding of **2AU-2** to a DNA hairpin that contains a stretch of four consecutive AT pairs was studied. Saturable binding was not observed (*K*_d_>10 μM; Supplementary Fig. 14). Interestingly, **AU-azide** binds to the DNA hairpin (EC_50_=116±15 nM) with similar affinity as the AU-rich RNAs (EC_50_=111±3 and 113±8 nM for AAUU and AUAU, respectively; Supplementary Figs 14 and 15). Thus, a monomeric binding module with modest selectivity can be reprogrammed to afford highly selective compounds for repetitive sequences by linking the binding modules together.

### Recognition of r(AUUCU)_500_ by 2AU-2 in cellular lysates

To investigate whether the designer dimer binds to r(AUUCU)_500_, we developed a method named Chem-Quant-Seq. To enable Chem-Quant-Seq a biotinylated derivative, **2AU-2-Biotin**, was synthesized (Fig. 7b and Supplementary Fig. 23). After incubation of 250 nM of **2AU-2-Biotin** with total RNA isolated from cells that express r(AUUCU)_500_ (ref. 33), bound RNAs were isolated with streptavidin resin. After extensive washing and elution of bound RNAs, the amount of r(AUUCU)_500_ bound to **2AU-2-Biotin** was quantified by using quantitative PCR with reverse transcription (qRT–PCR). Results show that there is a significant enrichment of the r(AUUCU)_500_ in the pulled-down material, as compared to 18S rRNA, showing that the compound indeed recognizes that target in the presence of cellular RNAs (Fig. 7c). To further assess the ability of the compound to recognize r(AUUCU)_500_ over shorter repeats of non-pathogenic length, various concentrations of r(AUUCU)_11_ in excess of r(AUUCU)_500_ were added to the lysate and enrichment of r(AUUCU)_500_ in the pulled-down fraction was quantified. Results from this competition experiment showed that 100-fold excess of r(AUUCU)_11_ was required to decrease enrichment of r(AUUCU)_500_ in the pulled-down fraction (Fig. 7d). Thus, **2AU-2-Biotin** recognizes long repeats over shorter ones. Furthermore, these data also point to cooperative binding of **2AU-2** to r(AUUCU) repeats as being a manner in which longer repeats are preferred over shorter ones. Positive cooperativity for binding to **2AU-2** was also observed *in vitro* (Hill coefficients; Table 2).

We further profiled target selectivity amongst 93 highly abundant transcripts in the pulled-down fraction via qRT–PCR. The RNAs span the diverse biology in the transcriptome, including rRNAs, mRNAs, small RNAs (sRNAs) and tRNAs. The rRNAs included all four rRNA subunits (18S, 28S 5S and 5.8S) and 45S rRNA. The 50 mRNAs were chosen from the most abundant mRNAs in HeLa cells for which there are established qRT–PCR primers^42^. The 17 sRNAs were selected from different structural and functional classes^43^: small nucleolar RNAs (HBII-85, HBII-420, U105 C/D Box snoRNAs, and ACA-16, ACA-44, ACA-61, HBI-36 H/ACA box snoRNAs), small cajal body-specific RNA (U87 scaRNA), small nuclear RNAs (U1, U2, U4, U5, U6 and U12 snRNAs), BC200 RNA, 7SK RNA and 7SL RNA. The 21 tRNAs profiled were randomly selected.

Only a fraction of these RNAs were enriched comparably to r(AUUCU)_500_, showing **2AU-2** it possesses reasonable selectivity for the desired target (Fig. 7e). Of the nine RNAs that show significant enrichment, most are tRNAs. Since **2AU-2** was shown to pull down tRNAs, we studied its effect on translation in two different ways in HeLa cells: (i) by transfecting a plasmid that encodes green fluorescent protein (GFP); and (ii) co-transfecting plasmids that encode GFP and r(AUUCU)_500_. GFP is a commonly used gene reporter in translational studies because of its visually identifiable characteristics^44^. After 24 h incubation, the fluorescence intensity of GFP was measured (Supplementary Fig. 16). Importantly, no change in the expression of GFP was observed in either system after treatment with **2AU-2**, as compared with an untreated control (Supplementary Fig. 16). Thus, **2AU-2** does not affect translation at its active concentrations where it improves SCA10-associated defects, 50 and 100 nM, *vide infra*. Interestingly, 40% of the amino acids in GFP are encoded by seven of the tRNAs pulled down by **2AU-2**. Taken together with the lack of toxicity in healthy and SCA10 patient-derived cells (*vide infra*), these data suggest that binding to tRNAs in a pull-down is not sufficient to elicit a biological effect.

### Bioactivity of 2AU-2 in SCA10 patient-derived fibroblasts

**Figure 8:**
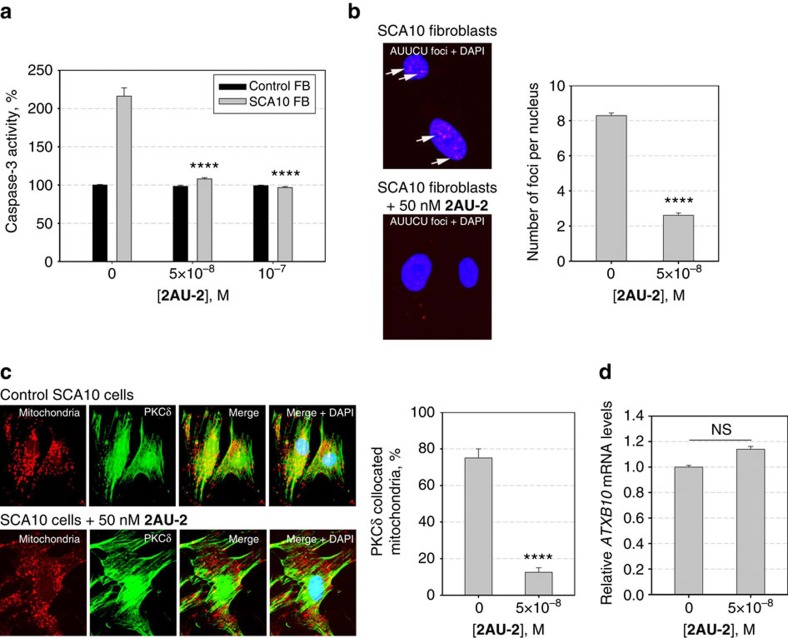
Studying the bioactivity of 2AU-2 in SCA10 patient-derived fibroblasts. (**a**) Relative caspase-3 activities in normal and SCA10 fibroblasts before and after treatment with compound **2AU-2**. Caspase-3 in SCA10 was reduced to normal levels when cells were treated with 50 nM compound. (**b**) Confocal images showing r(AUUCU)^exp^-containing nuclear foci in SCA10 cells without treatment (left, top) and after treatment with 50 nM **2AU-2** (left, bottom). Quantification of the number of r(AUUCU)^exp^-containing nuclear foci (right) in treated and untreated cells. (**c**) confocal images showing mitochondrial translocation of PKCδ after treatment with 50 nM **2AU-2** (left, bottom) compared with control, untreated SCA10 cells (left, top). Quantification of PKCδ collocated to mitochondria in treated and untreated cells (right). (**d**) Real-time RT–PCR analysis of *ATXN10* mRNA expression levels after treatment with **2AU-2**. The amount of mRNA was normalized relative to *GAPDH* mRNA. Values are reported as the mean±s.e. (*n*=6). ****P<0.0001 and ‘ns' denotes *P*>0.05, as compared with the untreated sample with a two-tailed Student *t*-test.

Encouraged by these results, the bioactivity of **2AU-2** was assessed by measuring the ability of the compound to improve three downstream disease-associated defects in SCA10 patient-derived fibroblasts^33^. As mentioned above, caspase 3 is abnormally activated in SCA10 fibroblasts by greater than twofold^33^. Thus, the effect of **2AU-2** on the caspase-3 activity in control and SCA10 fibroblasts was measured. When SCA10-affected cells were treated with 50 and 100 nM of **2AU-2** for 48 h, caspase-3 activity was reduced to levels observed in healthy cells (Fig. 8a). For comparison, we also tested the monomer **AU-azide** and two dimers with reduced *in vitro* potencies, **2AU-3 and 2AU-4**. In contrast to **2AU-2**, treatment with 100 nM **AU-azide** reduced levels of over-activated caspase-3 activity by only ∼30% while **2AU-3 and 2AU-4** were inactive up to 100 nM dosage despite showing activity *in vitro* (Supplementary Fig. 17). These results suggest that **2AU-3** and **2AU-4**, which have suboptimal distances between binding modules, may bind off-targets in cells. Thus, optimal bioactivity of **2AU-2** is a function of valency and the spacing between RNA-binding modules, which affords affinity and selectivity.

In agreement with the downregulated caspase-3 activity, **2AU-2** also reduced the mitochondrial abundance of PKCδ in SCA10 fibroblasts (Fig. 8c). PKCδ translocated to 70–80% of mitochondria in SCA10 cells, whereas only 10–15% mitochondria included PKCδ after treatment with 50 nM **2AU-2**, similar to levels observed in healthy fibroblasts. Next, the ability of **2AU-2** to disrupt formation of nuclear foci was measured. After 48 h incubation, **2AU-2** (50 nM) diminished ca. 70% of nuclear foci in SCA10 cells (Fig. 8b).

To confirm that improvement of SCA10-associated defects was due to binding of the compound to r(AAUCU)^exp^ and not toxicity, we studied the cytotoxicity of **2AU-2** in healthy and SCA10 fibroblasts by measuring released lactate dehydrogenase (LDH)^45^. No significant toxicity of the compound was observed at its active concentration, 50 nM, in either healthy or SCA10 fibroblasts (Supplementary Fig. 18). Thus, the observed downregulation of caspase-3 activity and reduction of the mitochondrial abundance of PKCδ in SCA10 fibroblasts does not result from compound toxicity. Likewise, these results are consistent with our observation that caspase-3 activity is unchanged in healthy fibroblasts upon **2AU-2** treatment.

Further, the observed bioactivity of **2AU-2** is not due to reduced abundance of the mutant *ATXN10* transcript carrying expanded AUUCU repeats as determined by qRT–PCR analysis (Fig. 8d). That is, the compound works at the RNA level, not at the transcriptional level. Interestingly, previous studies have shown that silencing of the *ATXN10* transcript improves SCA10-associated defects^33^ and some small molecules that improve microsatellite disease-associated defects work at the transcriptional level^46^.

Taken together, **2AU-2** markedly improves defects from hnRNP K sequestration by r(AUUCU)^exp^ in SCA10 patient-derived cells, suggesting that the compound binds to the cellular target, r(AUUCU)^exp^, and frees sequestered proteins. **2AU-2** is the most potent inhibitor known for a traditional non-covalent binder to repeat expansions.

## Conclusion

To identify selective RNA base pair binders, we screened small molecules that have RNA-binding scaffolds. The *bis*-benzamidine compound **2** is a selective AU base pair binder, as it binds AU base pairs 40-fold more strongly than GC base pairs. This compound was applied to target the pentanucleotide r(AUUCU) expansion that causes SCA10. The repeat periodically displays 5′AU/UA3′ base pair steps in its secondary structure. To improve affinity and selectivity, we modularly assembled **2** and determined that the optimal distance between RNA-binding modules was afforded by two propylamine spacing modules, or **2AU-2**. **2AU-2** significantly improves SCA10-associated defects to wild-type levels when patient-derived fibroblasts are treated with 50 nM compound. It is the first bioactive small molecule targeting r(AUUCU)^exp^. The potent bioactivity of **2AU-2** suggests that base pair-targeting RNA modules could have broad utility to provide bioactive compounds targeting other RNAs in the transcriptome.

## Methods

### Instrumentation

All pH measurements were performed at room temperature using a Mettler Toledo SG2 pH metre that was standardized at pH 4.0, 7.0, and 10.0. Absorption and emission spectra were measured using SpectraMax M5 plate reader (Molecular Devices, Inc.). Sigma Plot (version 11.0) was used for all curve fitting.

### Small molecules

All small modules were procured from the National Cancer Institute (NCI) or The Scripps Research Institute. Emission spectra (excitation: 300 nm, cutoff: 325 nm, emission: 330–600 nm) of all compounds were measured in a 384-well plate (Greiner Low-Volume 784076) to select fluorescent compounds for screening (50 μM compound in 1 × Screening Buffer (8 mM Na_2_HPO_4_, pH 7.0, 185 mM NaCl, 0.1 mM EDTA)).

### Compound purification and analysis

Preparative HPLC was performed using a Waters 1525 Binary HPLC pump equipped with a Waters 2487 dual-absorbance detector system and a Waters Sunfire C18 OBD 5-μm 19 × 150 mm column. Absorbance was monitored at 220 and 345 nm. A gradient of 20–100% methanol in H_2_O with 0.1% trifluoroacetic acid (TFA) over 60 min was used for compound purification. Analytical HPLC was performed using a Waters Symmetry C18 5 μm 4.6 × 150 mm column. Compounds were analysed using a gradient of 20–60% MeOH in H_2_O with 0.1% TFA over 30 min. All compounds evaluated had ≥95% purity as determined by analytical HPLC. Mass spectrometry was performed with an Applied Biosystems MALDI ToF/ToF Analyzer 4800 Plus and Microflex (Bruker) using an α-hydroxycinnamic acid matrix. See the Supplementary Information for details of compound synthesis and compound characterization.

### Oligonucleotide preparation and purification

The RNAs used in fluorescence binding assays, nuclease mapping, and filtre-binding assays were purchased from Dharmacon. The ACE protecting groups were cleaved by using Dharmacon's deprotection buffer by incubating at 60 °C for 30 min. The samples were lyophilized, resuspended in water and gel purified. Concentrations were determined by absorbance using a Beckman Coulter DU800 ultraviolet–visible spectrophotometer at 85 °C. Extinction coefficients (at 260 nm) were calculated using the HyTher server, which uses nearest-neighbour parameters^47^,^48^,^49^.

### Initial screen for small molecules that bind RNA base pairs

RNAs were folded in 1 × Screening Buffer at 95 °C for 2 min followed by slowly cooling to room temperature on the bench top. A 10-μl aliquot of a 1 μM RNA solution was dispensed into each well of a black 384-well plate (Greiner Low-Volume 784076) using an Aurora Discovery FRD-1B liquid dispenser. A 10-nl aliquot of a 2.5 mM stock of small molecule was pinned into each well using Biomek NXP Laboratory Automation Workstation that was equipped with a 384-pin head. The solution was incubated at room temperature for 30 min. Fluorescence intensity was measured using the maximum excitation/emission wavelength for each compound and the change in fluorescence was calculated by the ratio of *F*/*F*_0_ where *F* is the fluorescence intensity in the presence of RNA and *F*_0_ is the fluorescence intensity in the absence of RNA.

Compounds were scored as hits if a >20% change in emission (either enhancement or quenching) was observed upon incubation with RNA. The selectivity of a small molecule was computed by comparing the relative change in emission when incubated different RNAs. Statistically significant differences were calculated by using one-way analysis of variance function in Sigma Plot (version 11.0); compounds that had a *P* value of <0.05 (95% confidence) were chosen as selective binders.

### Chemoinformatic analysis

To identify the chemical substructures that facilitate binding, hit compounds were tested by an automated R-group analysis (Tripod Development; Division of preclinical Innovation, National Center for Advancing Translational Sciences: http://tripod.nih.gov/?p=46). The functional groups that provided recognition for each type of RNA were then compared.

### Fluorescence binding assays

Direct binding assays for all selective binders were performed. RNAs were folded as described above. Binding assays were performed with a constant compound concentration (1 or 3 μM) and serial dilutions of RNA or DNA in 1 × Screening Buffer. For molecular crowding experiments, PEG 8000 was added to a final concentration of 20% (w/v) to the folded RNA and to the solution used for serial dilutions. After a 20 min incubation, fluorescence intensity was measured. The resulting curves were fit to the following equation to determine EC_50_ values:


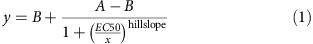


where *y* is fluorescence intensity, *x* is the concentration of RNA, *B* is the minimum fluorescence; *A* is the maximum fluorescence; and the EC_50_ is the concentration of RNA where half of the compound is bound.

Two types of plots were constructed to determine stoichiometries and *K*_d_'s: fluorescence versus [nucleic acid]/[ligand] to determine stoichiometry and fraction-bound/[nucleic acid] versus fraction bound to determine *K*_d_'s. Stoichiometries were determined from the former plots by fitting each of the two slopes (pre-saturated and saturated portions of the curves) to a line^50^. For unsaturated binding curves, the saturated portions of the curves were estimated by the fitted data from equation (1). The point at which the two equations intersect affords the stoichiometry. The *K*_d_'s were determined by fitting fraction bound/[nucleic acid] versus fraction bound to equation (2):


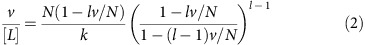


where *v* is the moles of RNA lattice bound per moles of ligand, [*L*] is the concentration of ligand, *N* is the number of repeating units on the RNA, *l* is the number of consecutive lattice units occupied by the ligand, and *k* is the microscopic dissociation constant.

### Nuclease mapping of the small molecule-binding site

r(AUUCU)_11_ was radioactively labelled at the 5′ end with [γ-^32^P] ATP (Perkin Elmer) and T4 polynucleotide kinase (New England Biolabs) using standard methods and gel purified to homogeneity^51^. The RNA was folded by incubation at 60 °C for 5 min in 1 × RNA Structure Buffer (Ambion) followed by slow cooling to room temperature. Serially diluted concentrations of the inhibitor was added to the RNA solution and incubated at room temperature for 15 min. RNase V1 (Ambion) was added to the RNA-inhibitor complex to a final concentration of 5 μU μl^−1^ and the samples were incubated at room temperature for 60 min. RNase V1 was then inactivated by heating at 95 °C for 1 min, and cleavage products were separated on a denaturing 20% polyacrylamide gel. A hydrolysis ladder was prepared by using Alkaline Hydrolysis Buffer (Ambion) and the manufacturer's protocol.

### Chemical syntheses of dimeric compounds

Details of compound syntheses and characterization are provided in Supplementary Figs 19–24 and Supplementary Table 4.

### Screening of dimeric compounds for binding to r(AUUCU)_11_ by filtre binding

To determine the optimal distance between RNA-binding modules, a library of dimers was synthesized and screened by using a filtre-binding assay. Radioactively labelled r(AUUCU)_11_ (100 nM) was folded in 1 × PBS buffer (pH 7.4) containing 1 mM MgCl_2_ by incubation at 60 °C for 5 min followed by slow cooling to room temperature. BSA was added to a final concentration of 50 μg ml^−1^ followed by addition of 1 μM compound. The samples were incubated at room temperature for 15 min. Nitrocellulose and nylon membranes were incubated in 1 × filtre binding assay buffer (1 × PBS buffer (pH 7.4) containing 1 mM MgCl_2_ and 50 μg ml^−1^ BSA). Bound and unbound RNA were separated using a Dot-Blot apparatus followed by washing with 1 × filtre binding assay buffer. The membranes were exposed to a phosphorimager screen and imaged using a Molecular Dynamics Typhoon phosphorimager. The amount of r(AUUCU)_11_ bound to each membrane was quantified using QuantityOne software (BioRad).

### Mobility shift assay of r(AUUCU)_11_ with DGCR8Δ

r(AUUCU)_11_ was 5′-end labelled as described above and folded in 1 × Folding Buffer (20 mM HEPES, pH 7.5, 110 mM KCl, and 110 mM NaCl) by incubation at 60 °C for 5 min followed by slow cooling to room temperature on the bench top. The buffer was adjusted to 1 × TR-FRET Assay Buffer (20 mM HEPES pH 7.5, 110 mM KCl, 110 mM NaCl, 0.1% BSA, 2 mM MgCl_2_, 2 mM CaCl_2_, 0.05% Tween-20 and 5 mM DTT) and various concentrations of DGCR8Δ were added. The samples were incubated at room temperature for 15 min and loaded onto a pre-chilled native 5% polyacrylamide gel. The gel was imaged and quantified as described above. The resulting curves were fit to equation (3):


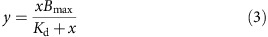


where *y* is percentage of bounded DGCR8Δ, *x* is the concentration of protein, *B*_max_ is maximum percentage of protein bound (restrained to equal 100%) and *k*_d_ is dissociation constant.

### Determination of compound potency via a TR-FRET assay

TR-FRET assays were completed as previously described^21^ with the following modifications. After folding the RNA, compound was added and incubated for 15 min at room temperature followed by addition of DGCR8Δ. The final concentrations of r(AUUCU)_11_ and DGCR8Δ were 60 and 40 nM, respectively. TR-FRET was measured after an additional 30-min incubation at room temperature. IC_50_ values were calculated by curve fitting using equation (1).

### Pull-down of **2AU-2**′s cellular targets

HeLa cells were maintained as monolayers in growth medium (1 × DMEM, 10% fetal bovine serum and 1 × GlutaMax (Invitrogen)). Cells were plated in 10-cm dishes and grown to ∼90% confluency and then transfected with a plasmid encoding (AUUCU)_500_ using Lipofectamine 2,000 (Invitrogen) per the manufacturer's recommended protocol. Cells were collected 18–24 h post-transfection and total RNA was extracted by using Trizol reagent (Ambion) according to the manufacturer's protocol. After RQ1 DNase (Promega) treatment, the DNase was removed by phenol:chloroform extraction and total RNA was ethanol precipitated. Next, 100 μg of total RNA was folded in 1 × Screening Buffer by heating at 75 °C for 5 min and cooling to room temperature slowly. The folded RNAs were incubated with **2AU-2-Biotin** for 30 min at room temperature. The solution was then incubated with streptavidin beads (250 μl of slurry, Sigma-Aldrich) for 30 min at room temperature with gentle shaking. The supernatant (containing unbound RNAs) was removed, and the beads were washed with 250 μl 1 × Screening Buffer after gentle shaking for 5 min at room temperature. Bound RNA was released from the beads by heating the beads in 300 μl H_2_O at 80 °C for 3 min twice. The solution containing bound RNAs was concentrated to 5–50 μl by vacuum concentration. Complementary DNA (cDNA) was generated from 40 ng of RNA using a qScript cDNA Synthesis Kit (Quanta Biosciences) per the manufacturer's protocol. qPCR was performed on an ABI 7900 HT Real-Time PCR System using the following primers to detect the r(AUUCU)_500_-containing RNA: 5′-AGTCTCTCTATGTTGCCCAGG-3′ and 5′-ACTTCCCGAAACACCGTCTC-3′. The relative fold enrichment of the RNA pulled by the compound was calculated by normalization to 18S rRNA.

### Profiling of cellular RNAs pulled down by **2AU-2-Biotin**

Profiling was completed by qRT-PCR as described above. The 93 highly abundant RNAs, including rRNAs, sRNA, tRNAs and mRNAs, were selected based on a previous report^42^. Primer sets for mRNAs were obtained from RTPrimerDB^52^ (www.rtprimerdb.org) and qPrimerDepot (http://primerdepot.nci.nih.gov). The DNA sequences of tRNAs were obtained from tRNAdb^53^ (http://trna.bioinf.uni-leipzig.de/) and primer sets were designed by using Primer 3 software (http://frodo.wi.mit.edu/primer3/). All sequences of primers are listed in Supplementary Table 3.

### Cell culture of SCA10 fibroblasts

SCA10 fibroblasts^33^ were cultured in MEM with Eagle–Earle salt and 2 mM L-glutamine containing 15% fetal bovine serum and antibiotic in 5% CO_2_ at 37 °C in 75-cm^2^ flasks. Compounds were dissolved in 50% DMSO and added to the SCA10 cells at the indicated concentrations. Fresh medium with drug was added to the cells after every 12 h and cells were collected after 48 h for the caspase-3 assay, detection of AUUCU RNA foci, or for analysing the subcellular translocation of protein kinase C δ (PKCδ).

### Translocation of PKCδ after drug treatment

SCA10 fibroblasts (2 × 10^4^ cells) were seeded in chamber slides. When cells were 80–90% confluent, fresh cell culture medium containing compound was added to the cells and incubated for 48 h. The drug-treated and control cells were then incubated with mitotracker deep red 633 (Invitrogen, USA) at a concentration of 250 nM in cell culture medium and incubated at 37 °C for 30 min. The cells were then washed three times with ice-cold 1 × PBS, fixed with 4% paraformaldehyde for 30 min at room temperature, washed three times with 1 × PBS and stored in 70% ethanol for up to 24 h. Cells were blocked with DAKO antibody blocking solution (serum free) and later double stained with anti-PKCδ 1:500 in DAKO antibody diluent. Goat anti-mouse 488 was used to identify PKCδ. Fluorescent photomicrographs were taken using an Hamamatsu Camera Controller using DP controller software.

### Fluorescent *in situ* hybridization to detect AUUCU RNA foci

SCA10 and control fibroblasts (2 × 10^4^ cells) were seeded in chamber slides. When the cells were 60–70% confluent, cell culture medium containing compound was added to the cells and incubated for 48 h. After incubating with compound, the cells were fixed with 4% paraformaldehyde for 30 min at room temperature, and washed three to four times with ice-cold 1 × PBS. The AUUCU RNA foci were detected using a Cy3-labelled (AGAAU)_10_ RNA oligonucleotide probe as described previously^33^. In brief, the control and SCA10 cells were pre-hybridized at 65 °C in RNA Hybridization Buffer for 1.5 h, and hybridized overnight in hybridization buffer containing 250 ng of (AGAAU)_10_ Cy3-labelled RNA oligo at 45 °C. Slides were rinsed with 1 × PBS three times and extensively washed 4 × 5 min to remove all non-specific binding of Cy3-labelled RNA probes. Slides were then mounted with DAPI mounting medium and fluorescent images were taken using a confocal microscope.

### Cytotoxicity of **2AU-2**

SCA10 and healthy fibroblasts were treated either with **2AU-2** (50, 100 and 500 nM) or vehicle for 24 h. Later, the culture medium containing LDH was collected and the amount of LDH in the medium was quantified using TOX7 cell toxicity assay kit (Sigma-Aldrich).

### Data availability

Data supporting the findings of this study are available within the article and its supplementary information files and from the corresponding author upon request.

## Additional information

**How to cite this article:** Yang, W.-Y. *et al*. Design of a bioactive small molecule that targets r(AUUCU) repeats in spinocerebellar ataxia 10. *Nat. Commun.* 7:11647 doi: 10.1038/ncomms11647 (2016).

## Supplementary Material

Supplementary Information Supplementary Figures 1-24, Supplementary Tables 1-4 and Supplementary References

## Figures and Tables

**Table 1:** EC_50_s (nM) and Hill coefficients (shown in parentheses) of compounds binding to the base paired RNAs shown in Fig. 1.

Compound	AAUU	AUAU	GGCC	GCGC
1*	170±10 (3.1±0.79)	45±1 (1.5±0.32)	390±20 (1.4±0.34)	240±10 (0.92±0.34)
2†	170±40 (1.5±0.24)	190±20 (1.6±0.28)	8,550±250 (0.85±0.08)	6,950±250 (0.77±0.16)
AU-azide†	45±6 (1.0±0.17)	31±4 (1.0±0.13)	>6,000	>2,000

^*^[Compound]=3 μM.

^†^[Compound]=1 μM.

**Table 2:** *K*_d_s, Hill coefficients (shown in parentheses) and stoichiometries of 2, AU-azide and 2AU-2 binding to RNA repeats.

RNA repeats	r(AUUCU)_11_		r(CUG)_12_*		r(CGG)_12_*		r(CCUG)_12_*	
Compound	*K*_d_, nM	Stoichiometry	*K*_d_, nM	Stoichiometry	*K*_d_, nM	Stoichiometry	*K*_d_, nM	Stoichiometry
**2**	300±47 (1.7±0.2)	11±0.5	∼4,000	∼7	∼2,000	∼6	∼4,000	∼11
**AU-azide**	375±34 (1.3±0.1)	13±0.9	∼5,000	∼11	∼3,000	∼9	∼5,000	∼16
**2AU-2**	185±1.0 (1.4±0.4)	7±0.1	∼4,000	∼9	∼4,000	∼6	∼4,000	∼6

^*^The stoichiometry from unsaturated binding curves was estimated from data fit by equation (1). The *K*_d_ was calculated by using the estimated stoichiometry.
