# Supplementary material for: Design of a bioactive small molecule that targets r(AUUCU) repeats in spinocerebellar ataxia 10
Source: Nat Commun. 2016 Jun 1;7:11647. doi: 10.1038/ncomms11647 (PMC4895354; doi:10.1038/ncomms11647)
Supplement: Supplementary Information — Supplementary Figures 1-24, Supplementary Tables 1-4 and Supplementary References [file ncomms11647-s1.pdf]

Supplementary Figures

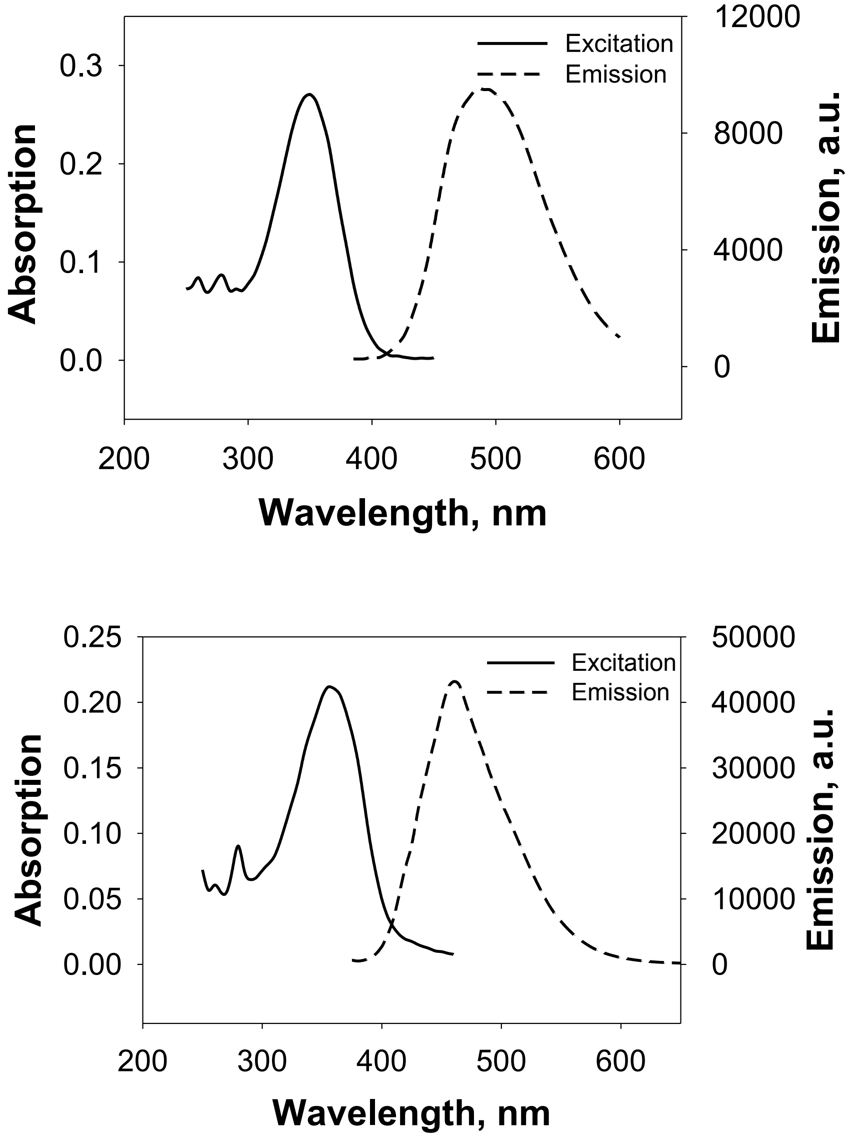

Supplementary Figure 1. Excitation and emission spectra of compound **1** (top) and **2** (bottom).

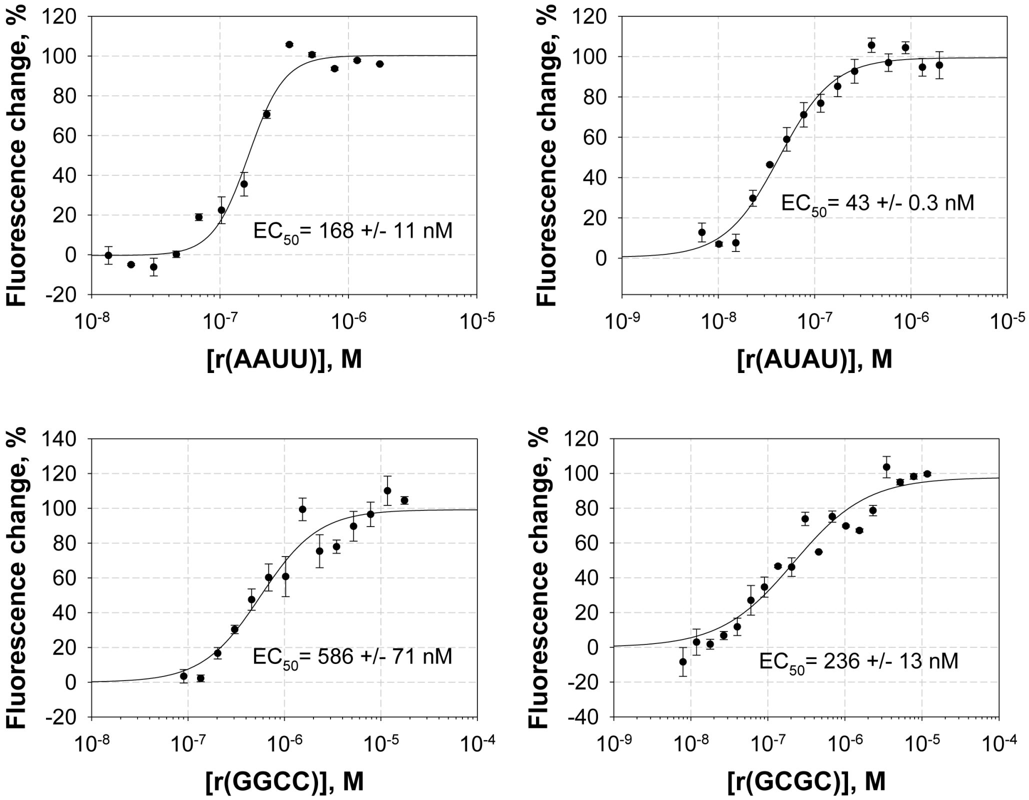

Supplementary Figure 2. Representative binding curves for compound **1** and r(AAUU), r(AUAU), r(GGCC) or r(GCGC) stretches ([**1**]= 3 μM).

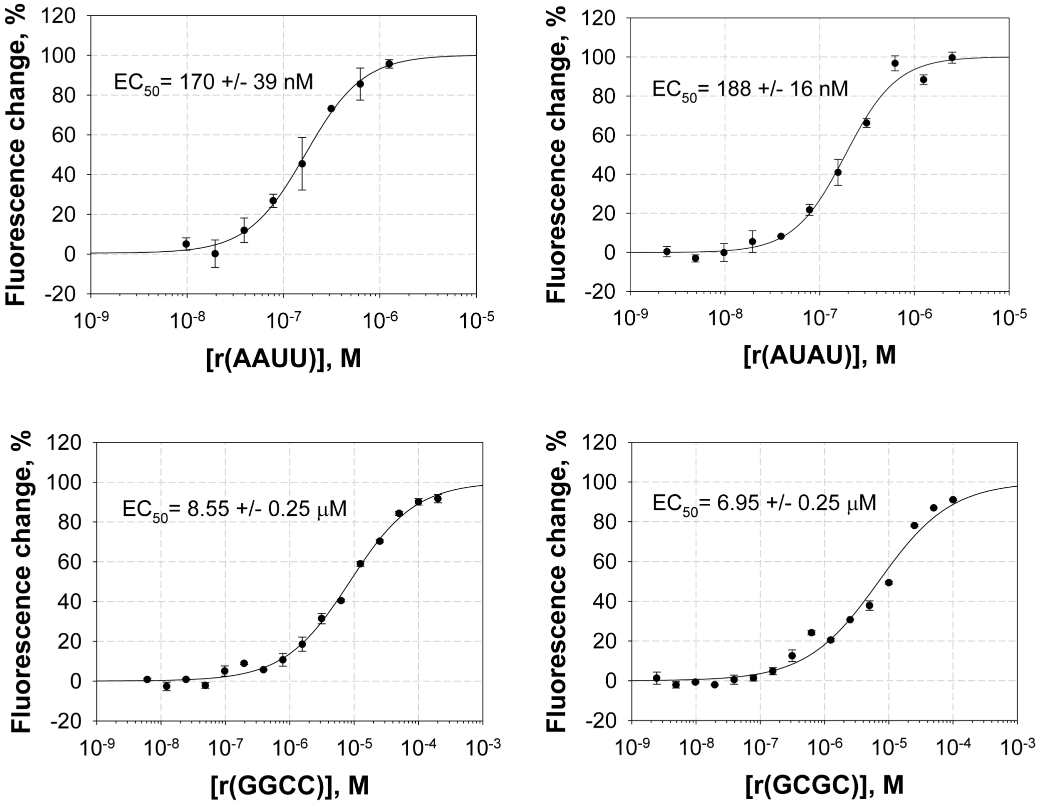

Supplementary Figure 3. Representative binding curves for compound **2** and r(AAUU), r(AUAU), r(GGCC) or r(GCGC) stretches ([**2**]= 1 μM).

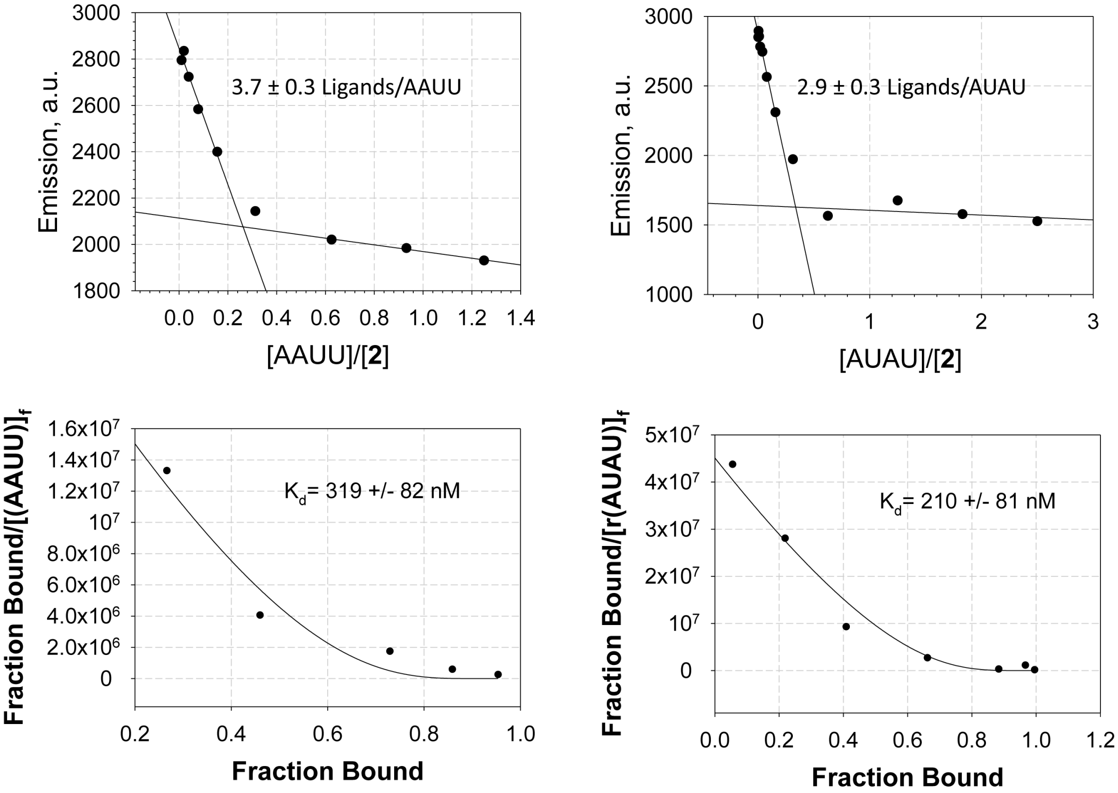

Supplementary Figure 4. Stoichiometries and K_ds for compound **2** binding to r(AAUU) and r(AUAU)stretches.

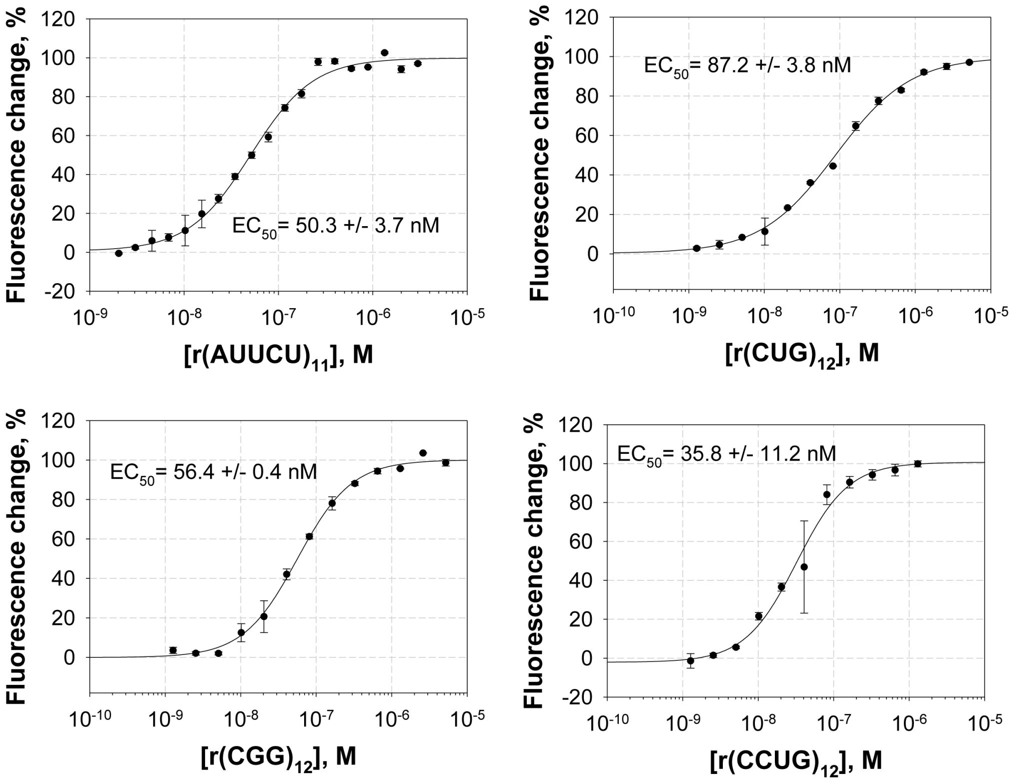

Supplementary Figure 5. Representative binding curves for compound **1** and r(AUUCU)$_{11}$, r(CUG)$_{12}$, r(CGG)$_{12}$ and r(CCUG)$_{12}$ ([**1**]= 3 μM).

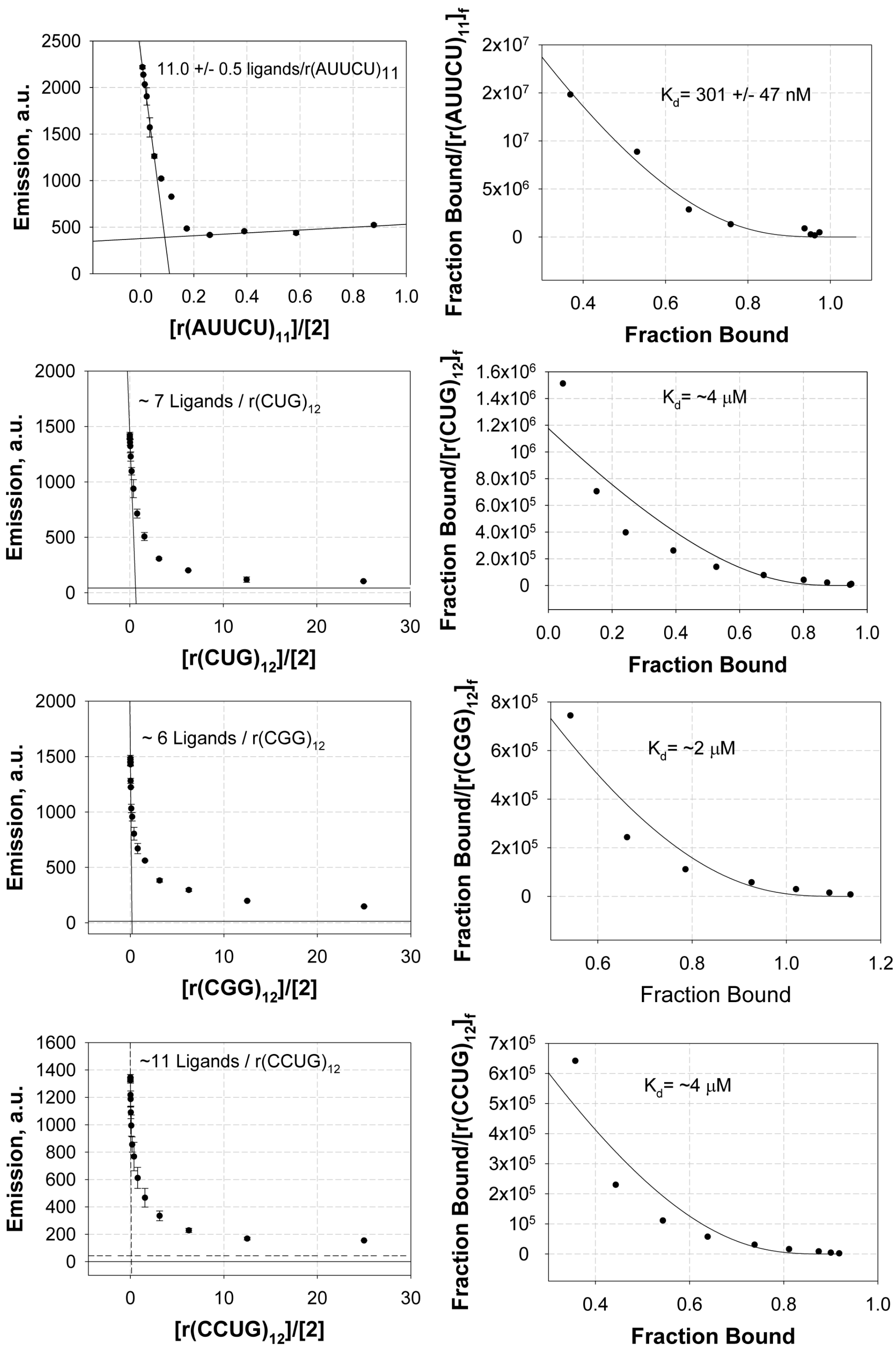

Supplementary Figure 6. Binding data (stoichiometries and K_ds) for **2** binding to r(AUUCU)$_{11}$, r(CUG)$_{12}$, r(CGG)$_{12}$ and r(CCUG)$_{12}$ ([**2**]= 1 μM).

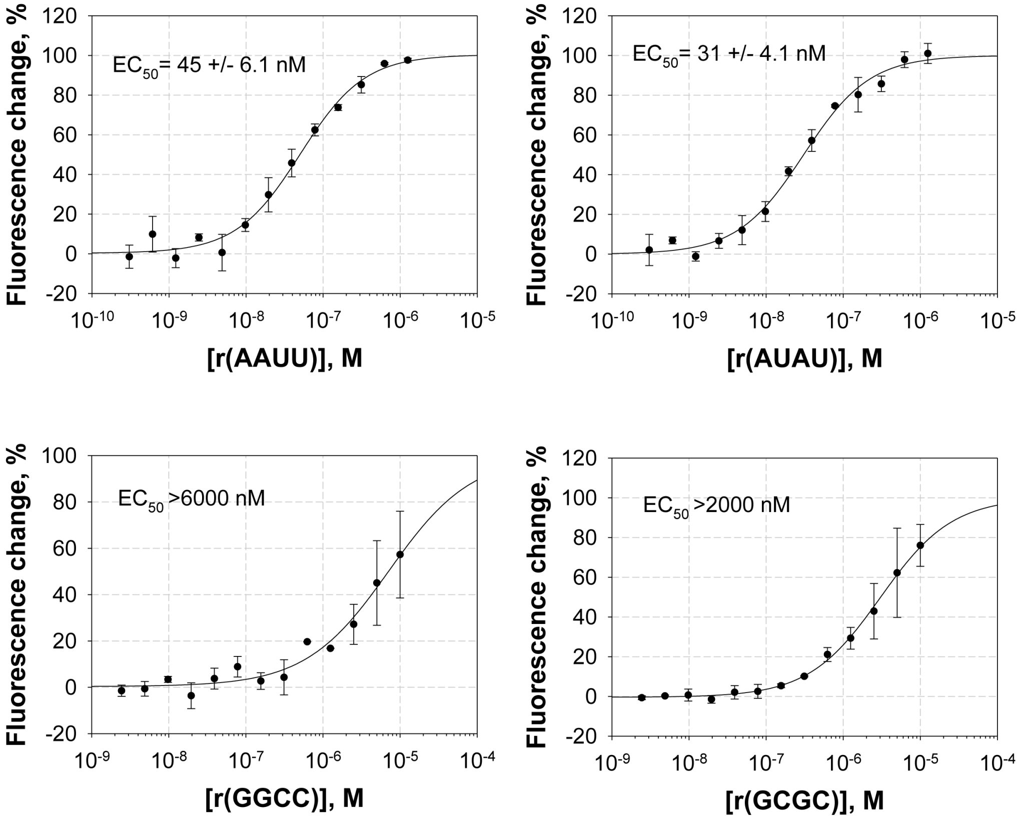

Supplementary Figure 7. Representative binding curves for compound **AU-azide** and r(AAUU), r(AUAU), r(GGCC) or r(GCGC) stretches ([**AU-azide**]= 1 μM).

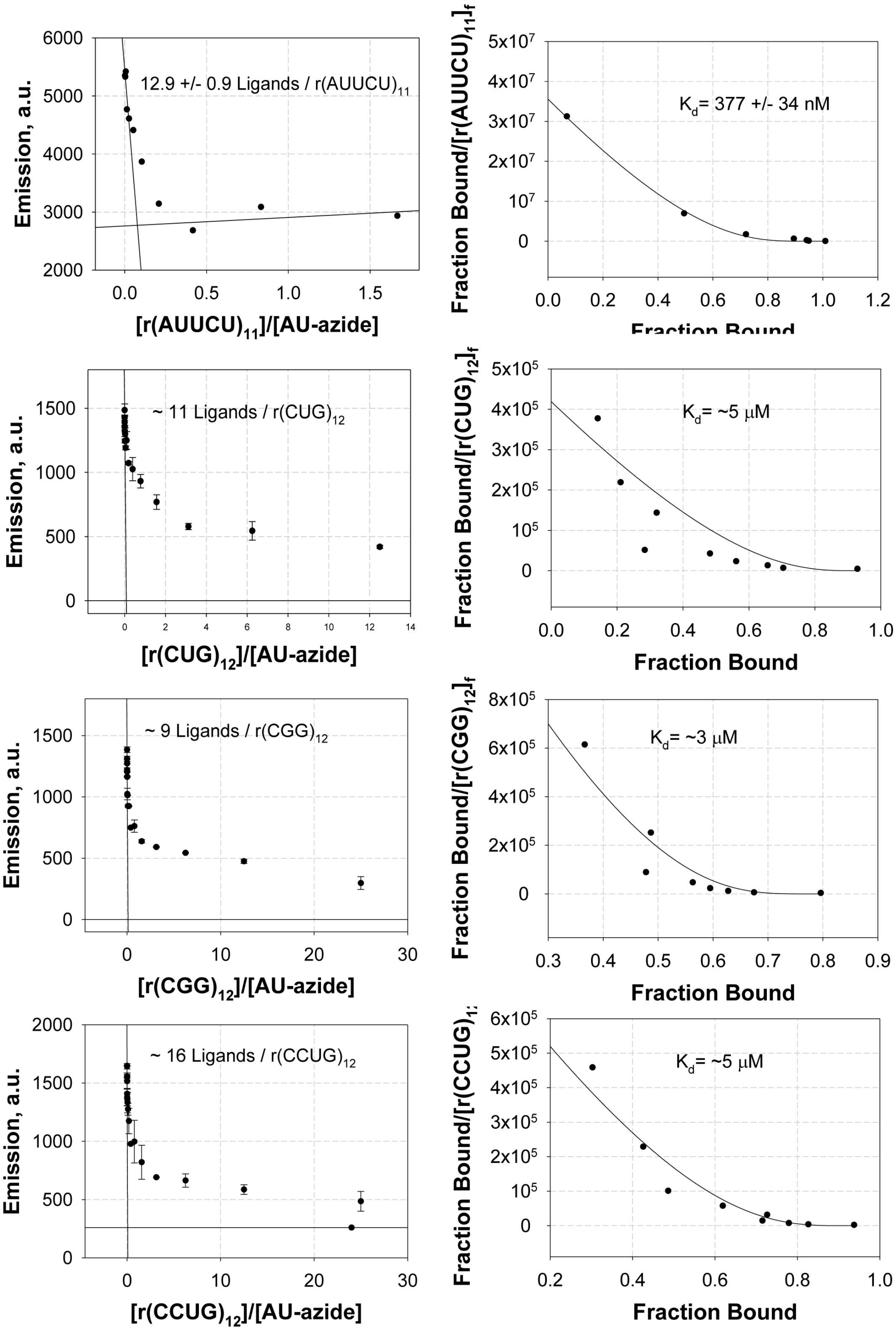

Supplementary Figure 8. Binding data (stoichiometry and K_d) for **AU-azide** binding to $r(AUUCU)_{11}$, $r(CUG)_{12}$, $r(CGG)_{12}$ and $r(CCUG)_{12}$ ([**AU-azide**]= 1 μM).

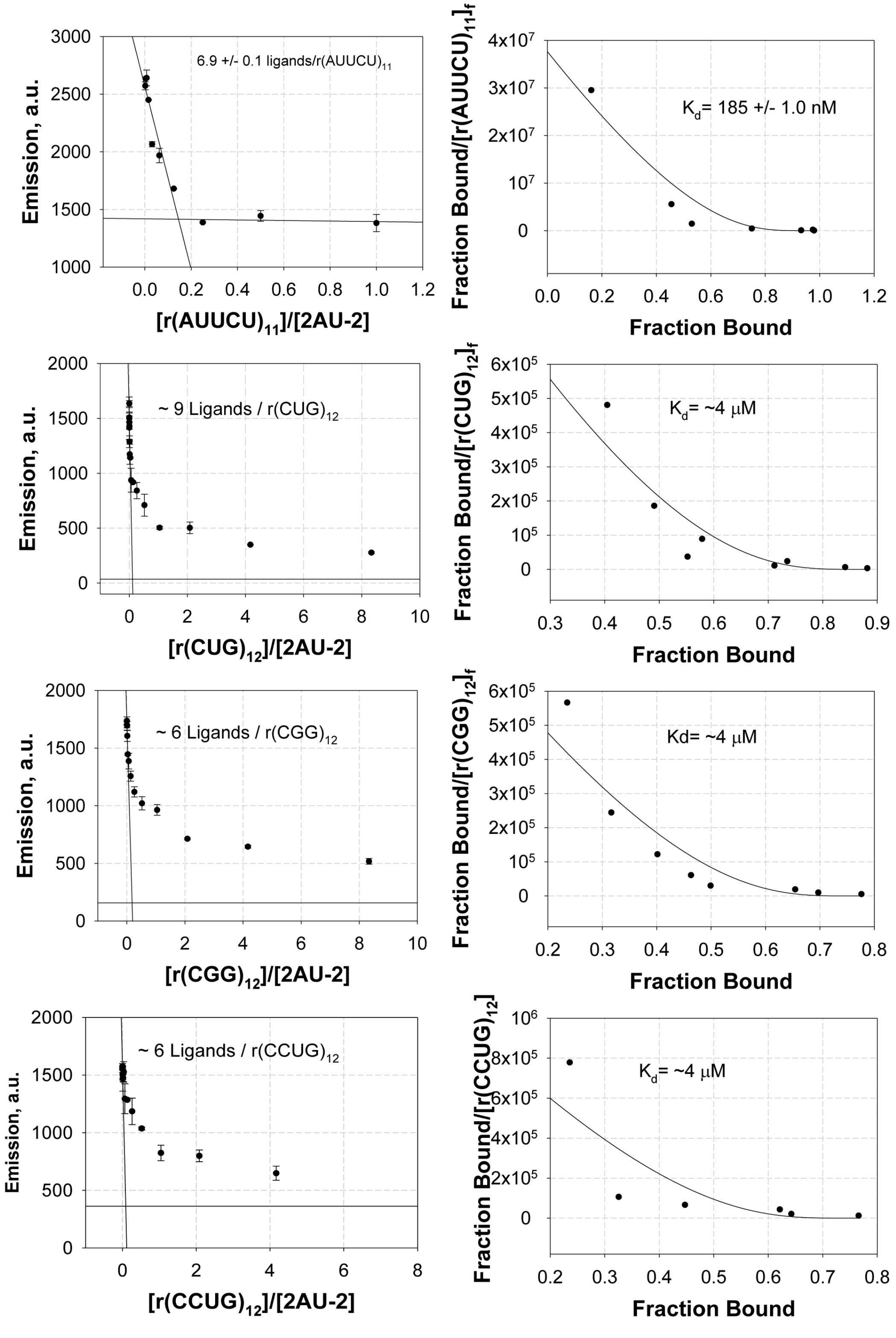

Supplementary Figure 9. Binding data (stoichiometries and K_ds) for compound **2AU-2** binding to r(AUUCU)$_{11}$, r(CUG)$_{12}$, r(CGG)$_{12}$ and r(CCUG)$_{12}$ ([**2AU-2**]= 3 μM).

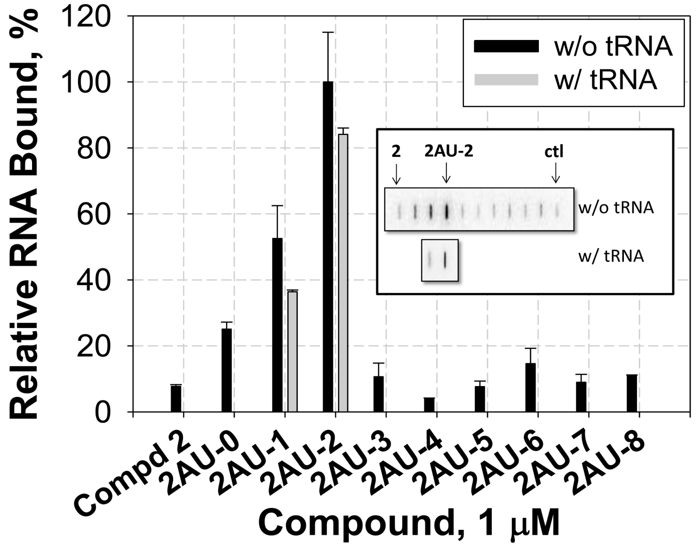

Supplementary Figure 10. Quantification of the filter binding assay results for screening dimeric compounds and images of nitrocellulose membranes (inset). Where indicated, 35-fold molar excess of tRNA was used (gray bars).

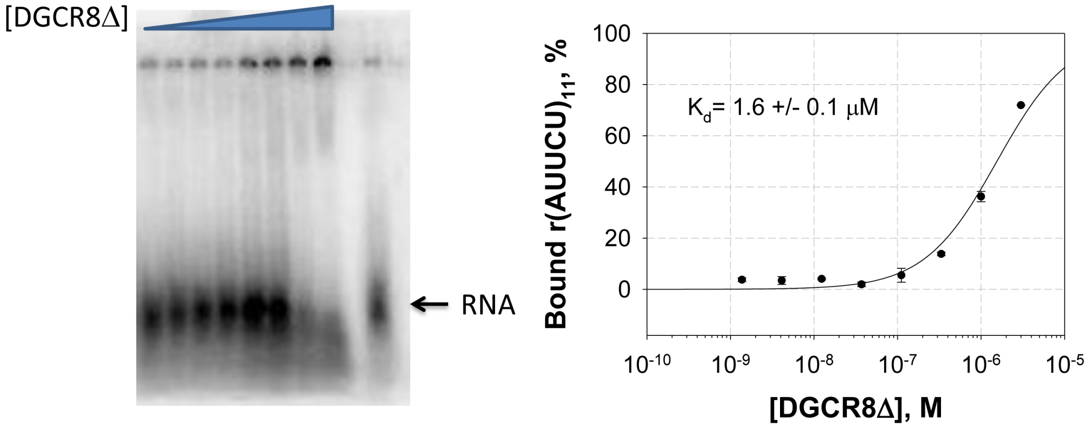

Supplementary Figure 11. Representative gel image of the binding of r(AUUCU)$_{11}$ to DGCR8Δ (left) and its quantification.

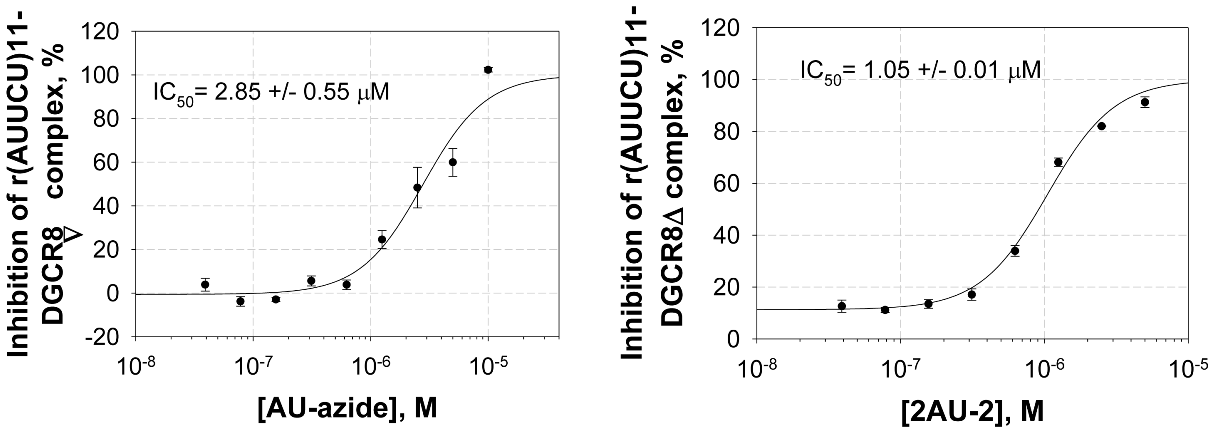

Supplementary Figure 12. IC_{50} of **2AU-2** and **AU-azide** for inhibiting binding of DGCR8Δ.

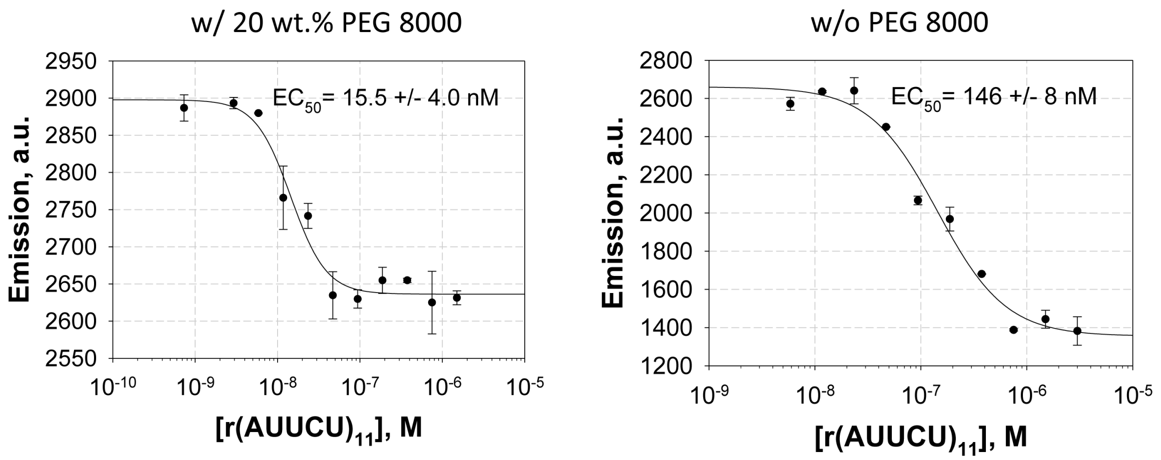

Supplementary Figure 13. Binding of **2AU-2** to r(AUUCU)$_{11}$ under molecularly crowded conditions.

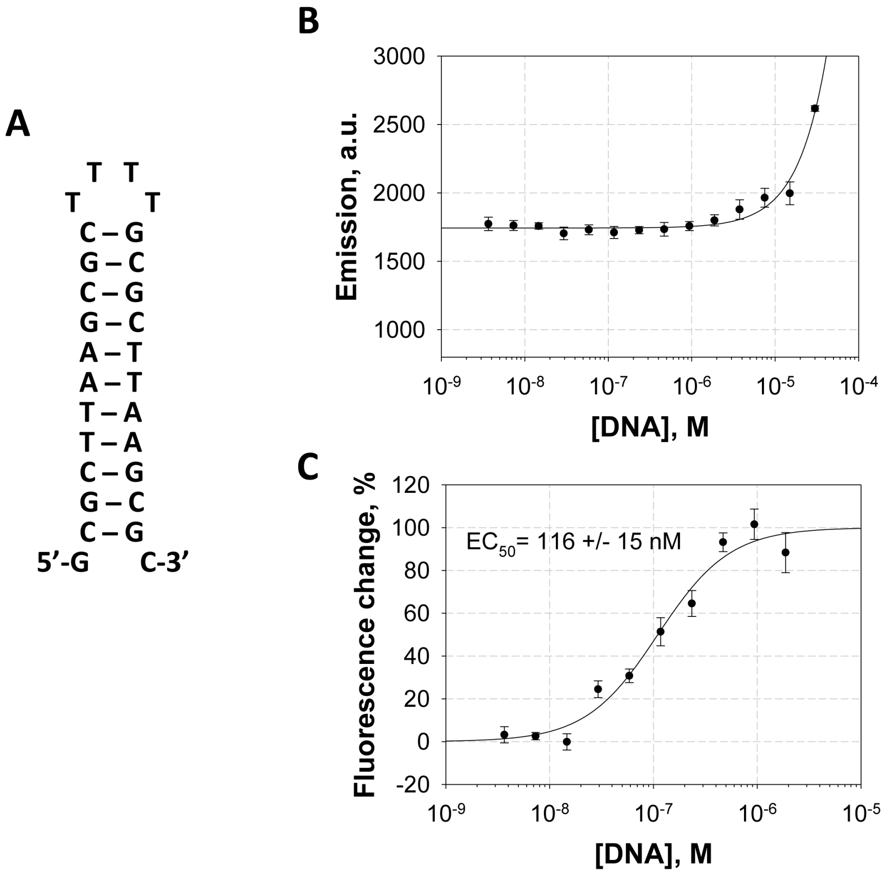

Supplementary Figure 14. The secondary structure of DNA (left) and the binding titration curve of **2AU-2** to the DNA (right). Saturable binding for **2AU-2** is not observed. [Compound]= 3 μM.

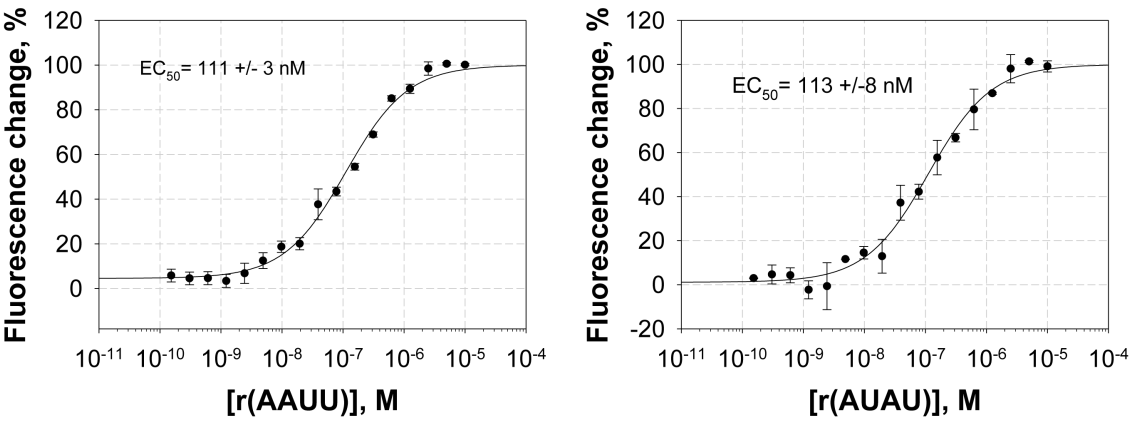

Supplementary Figure 15. EC_{50}s of **AU-azide** to r(AAUU) and r(AUAU). [Compound]= 3 μM.

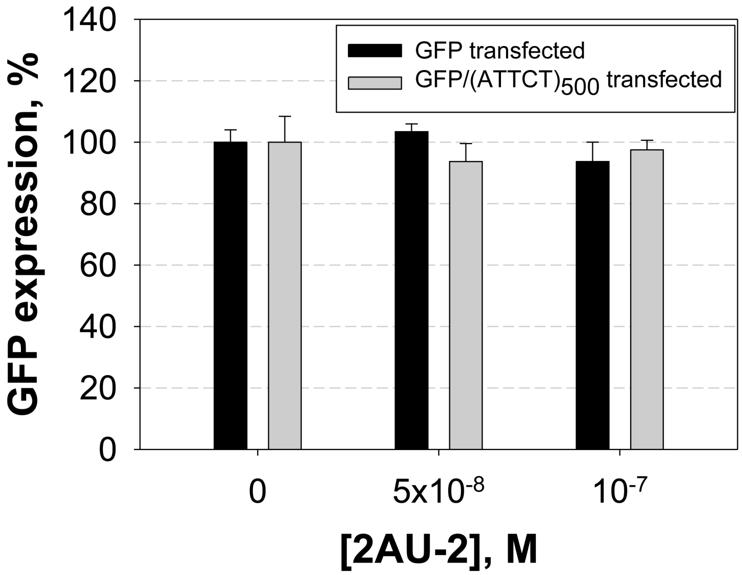

Supplementary Figure 16. Relative green fluorescent protein (GFP) expression measured by GFP fluorescence upon exposure to **2AU-2** compound. These data show that the compound has no effect on translation.

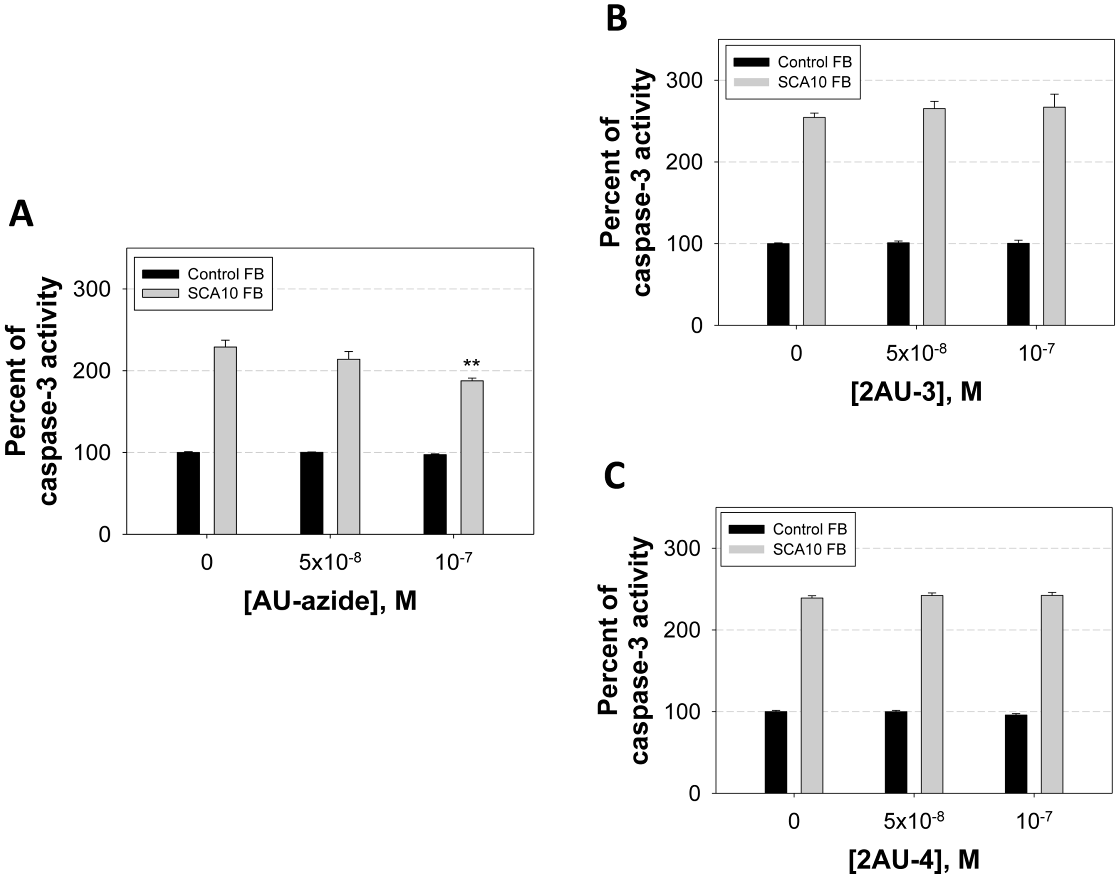

Supplementary Figure 17. Relative caspase-3 activities in normal and SCA10 fibroblasts before and after treatment with control compounds. **A**, **AU-azide**; **B, 2AU-3**; and **C, 2AU-4**.

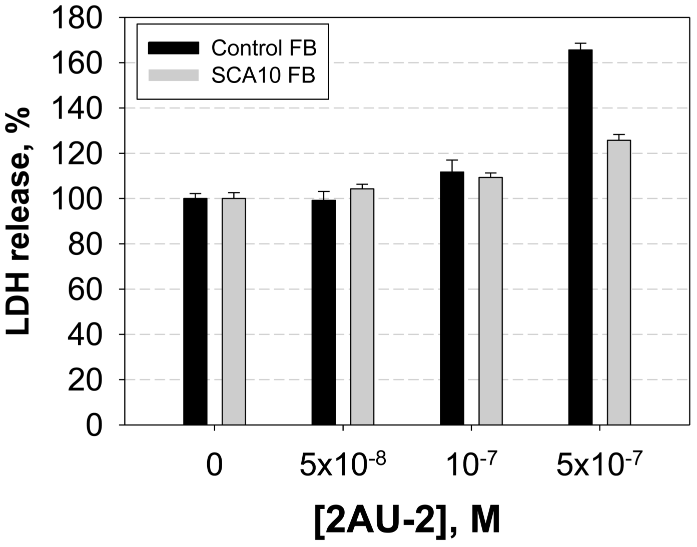

Supplementary Figure 18. Cytotoxicity of **2AU-2** by measuring released LDH.

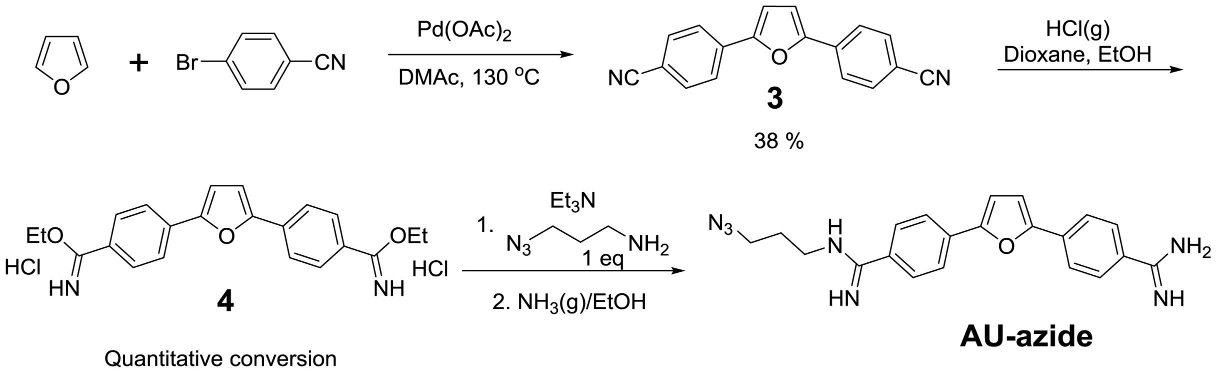

Supplementary Figure 19. Synthesis of **AU-azide**.

4,4'-(Furan-2,5-diyl)dibenzonitrile (3). To a mixture of 4-bromobenznitrile (5.5 mmoles) in 20 mL N,N-dimethylacetamide (DMAc) in a sealing tube were added furan (2.8 mmoles), KOAc (11 mmoles) and 0.5% $Pd(OAc)_2$. The reaction was stirred at 130 °C overnight and the solvent was removed under reduced pressure. The product (38% yield) was isolated by flash silica gel column chromatography with 6-40% ethyl acetate (EtOAc)/ hexanes (Hex) of eluent solvent.[1]

Diethyl 4,4'-(furan-2,5-diyl)dibenzimidate hydrochloride (4). 4,4'-(Furan-2,5-diyl)dibenzonitrile was dissolved in the mixture of 1,4-dioxane and ethanol (EtOH) and HCl gas was bubbled into the solution at 0 ^{o}C for X h. The solvent was removed under vacuo and the product was obtained in quantitative yield.[2]

***N*-(3-Azidopropyl)-4-(5-(4-carbamimidoylphenyl)furan-2-yl)benzimidamide (AU-azide).** Diethyl 4,4'-(furan-2,5-diyl)dibenzimidate hydrochloride (2.1 mmoles) and Et_3N (5.25 mmoles) were dissolved in 25 mL EtOH and 3-azidopropylamine (2.1 mmoles) in 25 mL EtOH was added dropwise over 30 min. The mixture was stirred at room temperature overnight. Then, NH_3 gas was bubbled into the solution at 0 °C for 3 h. The solution was transferred to a sealing tube and

stirred at room temperature for 4 days. After removing the solvent under vacuo, the product was isolated by preparative HPLC (10-100% methanol (MeOH)/H_2O with 0.1% trifluoracetic acid (TFA) for 60 min.). ^{1}H-NMR (400 MHz, DMSO-d_6) δ 1.93 (2Hs, tt, *J*= 6.8 Hz, *J*= 6.4 Hz), 3.47 (2Hs, t, *J*= 6.4 Hz), 3.52 (2Hs, t, *J*= 6.8 Hz), 7.46 (2Hs, m), 7.85 (2Hs, d, *J*= 8.8 Hz), 7.93 (2Hs, d, *J*= 8.4 Hz), 8.11 (4Hs, d, *J*= 8.4 Hz), 9.08 (1H, s), 9.17 (1H, s), 9.36 (1H, s), 9.53 (1H, s), 9.79 (1H, m); ^{13}C-NMR (400 MHz, DMSO-d_6) δ 26.58, 48.20, 111.56, 111.75, 123.80, 123.84, 126.70, 127.71, 128.98, 129.06, 133.87, 134.28, 152.37, 152.49, 162.70, 164.83; HRMS (ESI$^+$) calculated for $C_{21}H_{22}N_7O$: 388.1886, found: 388.1876.

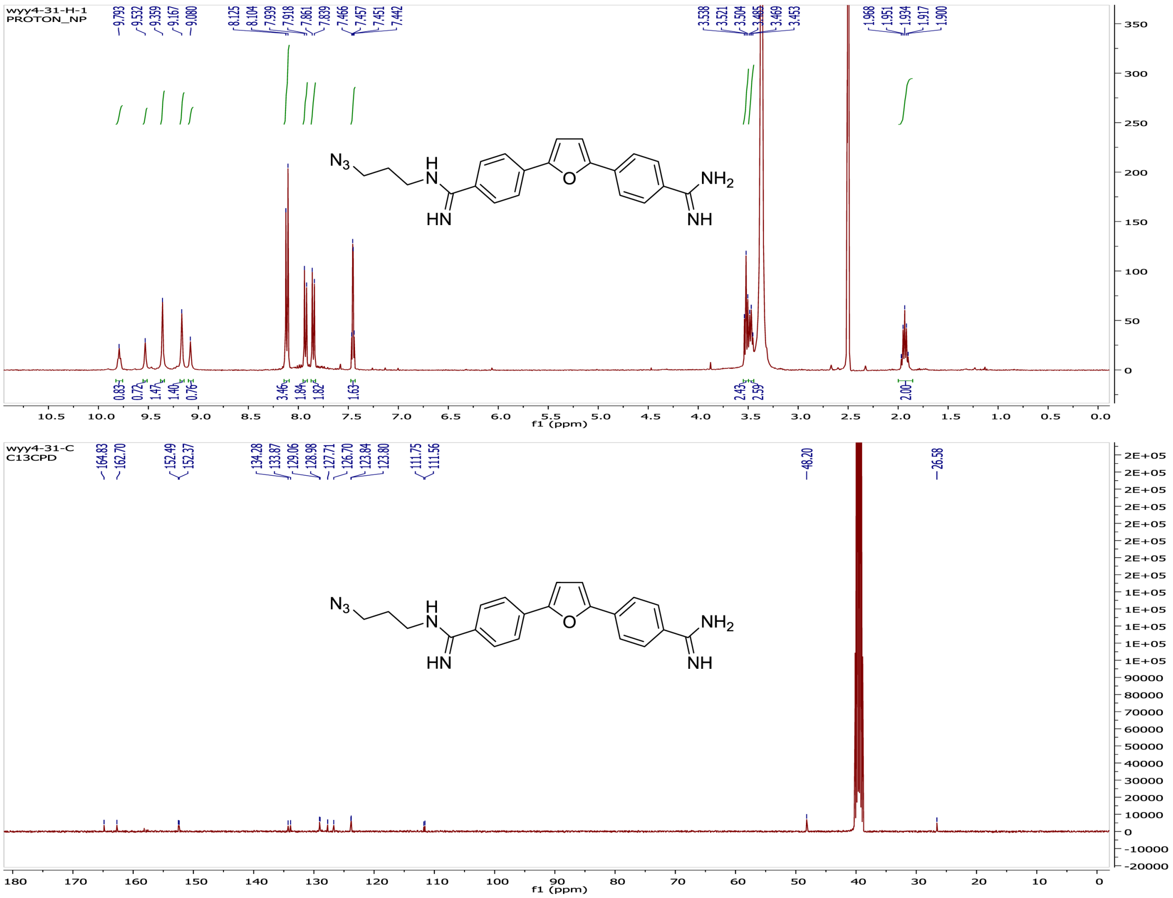

Supplementary Figure 20. ^{1}H and ^{13}C-NMR spectra of **AU-azide**.

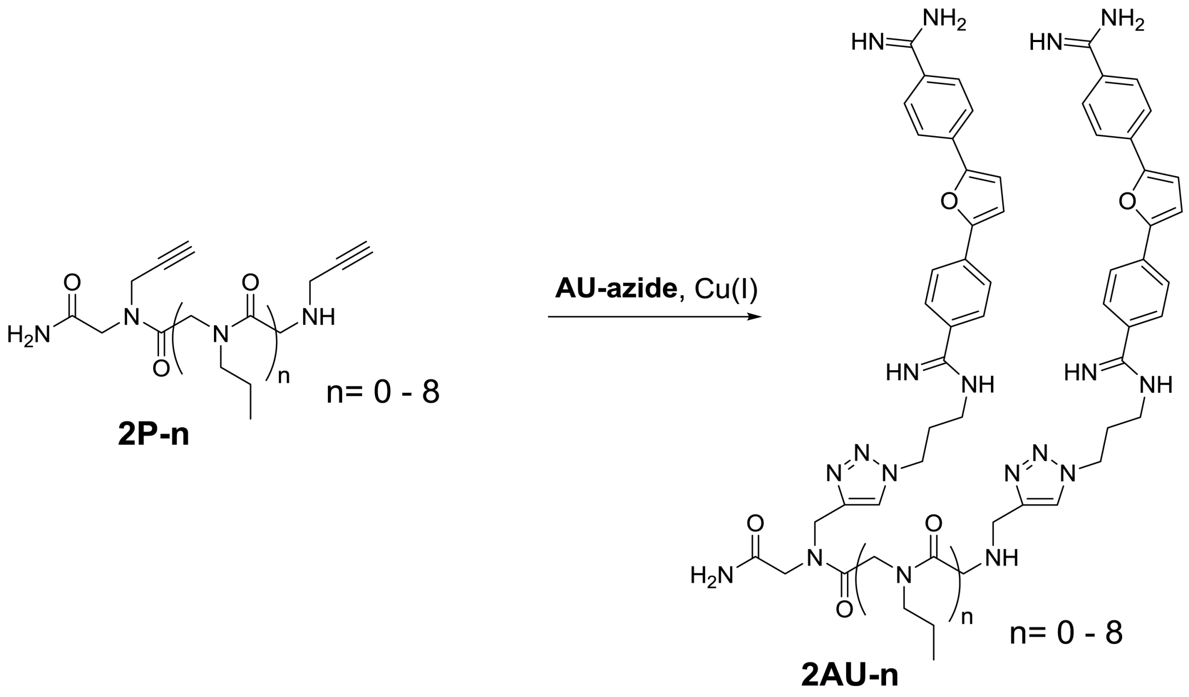

Supplementary Figure 21. Syntheses of **2AU-n**.

*General procedure for **AU-azide** conjugation to peptoids.* The corresponding peptoid backbone, synthesized as previously described,[3] was dissolved in DMF and Cu(I) catalyst, *N*,*N*-diisopropylethylamine (DIPEA) and **AU-azide** were added to the solution. The mixture was stirred overnight and the conjugate was purified by using reverse phase HPLC using a linear gradient of 20-100% MeOH in H_2O with 0.1% TFA over 30-60 min.

HPLC traces and mass spectra for modularly assembled compounds

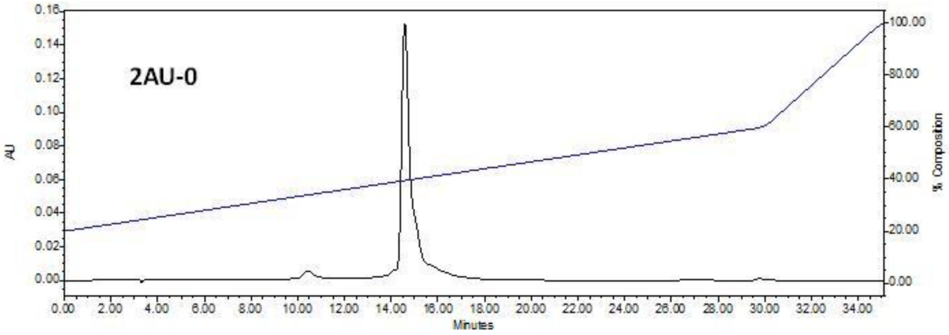

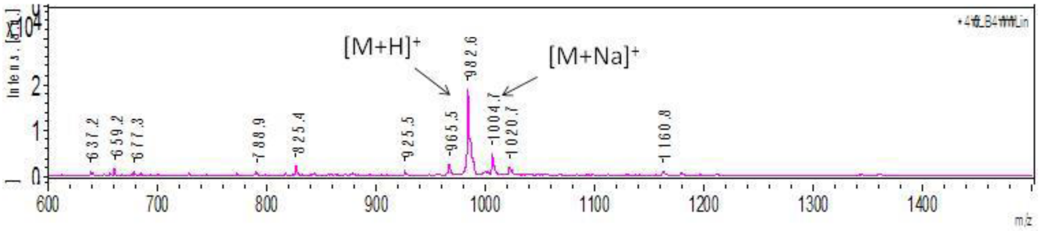

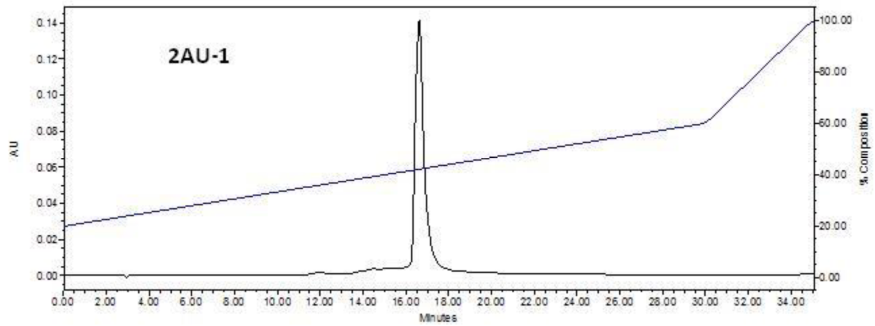

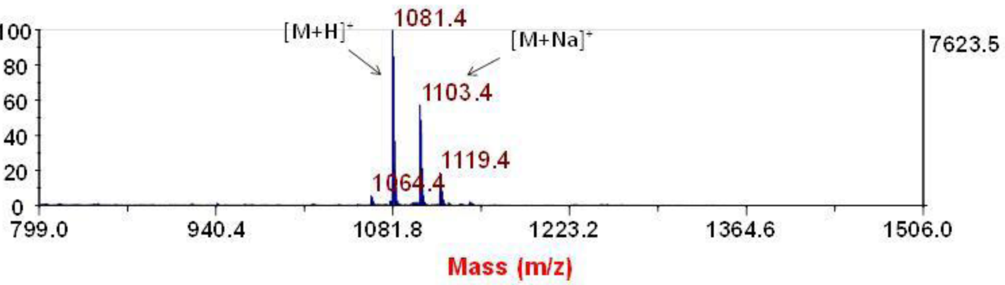

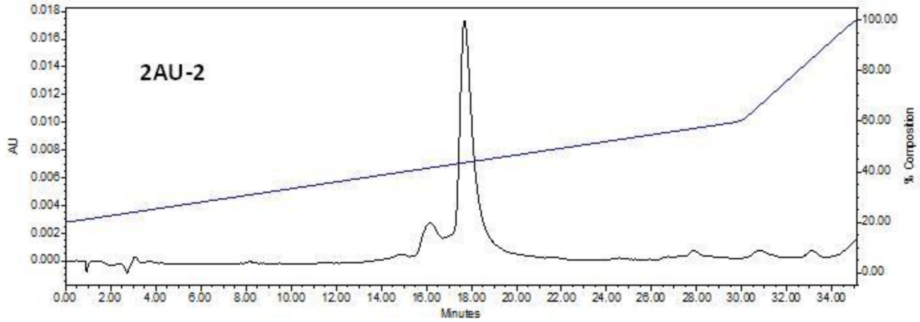
2AU-2
AU
% Composition
Minutes
0.018
0.016
0.014
0.012
0.010
0.008
0.006
0.004
0.002
0.000
100.00
80.00
60.00
40.00
20.00
0.00
0.00 2.00 4.00 6.00 8.00 10.00 12.00 14.00 16.00 18.00 20.00 22.00 24.00 26.00 28.00 30.00 32.00 34.00

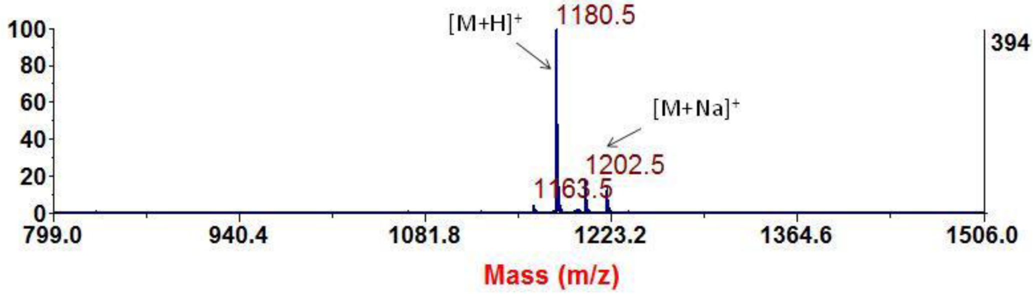
[M+H]+
1180.5
[M+Na]+
1202.5
1163.5
394
100
80
60
40
20
0
799.0 940.4 1081.8 1223.2 1364.6 1506.0
Mass (m/z)

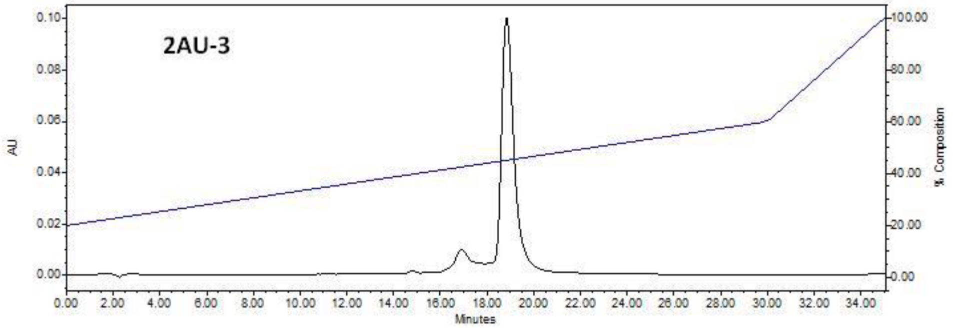
2AU-3
AU
% Composition
Minutes
0.10
0.08
0.06
0.04
0.02
0.00
100.00
80.00
60.00
40.00
20.00
0.00
0.00 2.00 4.00 6.00 8.00 10.00 12.00 14.00 16.00 18.00 20.00 22.00 24.00 26.00 28.00 30.00 32.00 34.00

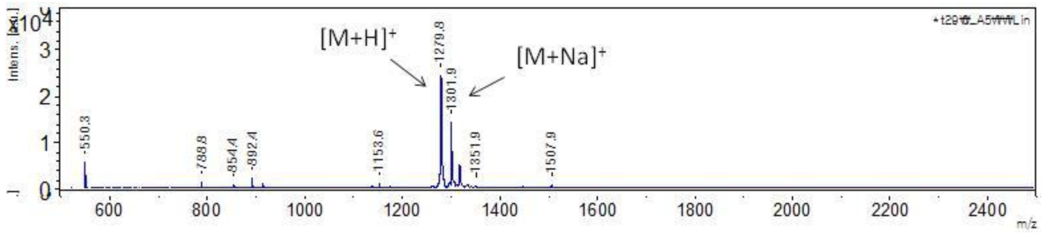
Intens. [a.u.]
x10^4
[M+H]+
[M+Na]+
1279.8
1301.9
550.3
789.8
854.4
892.4
1153.6
1351.9
1507.9
600 800 1000 1200 1400 1600 1800 2000 2200 2400
m/z

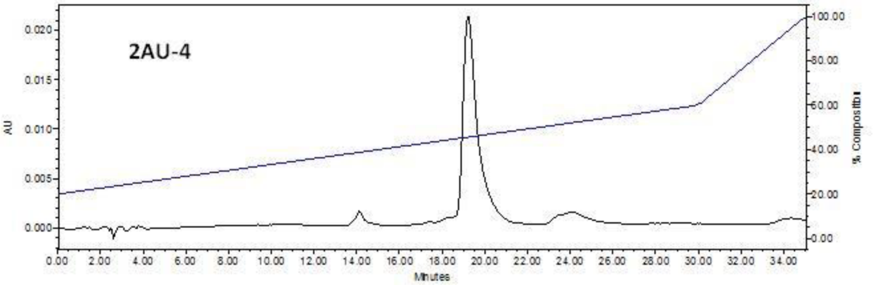
2AU-4
AU
% Composition
Minutes

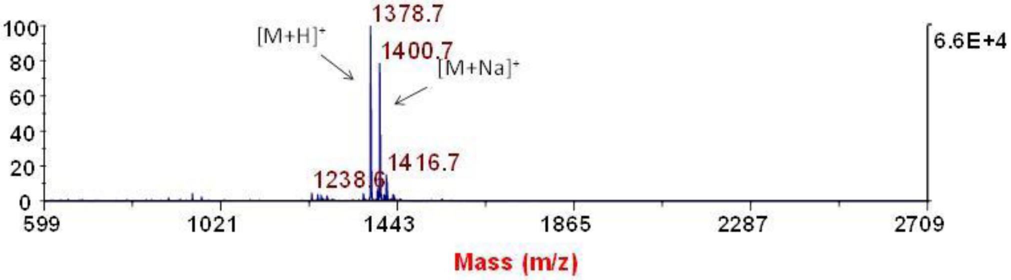
1378.7
[M+H]+
1400.7
[M+Na]+
1416.7
1238.6
6.6E+4
Mass (m/z)

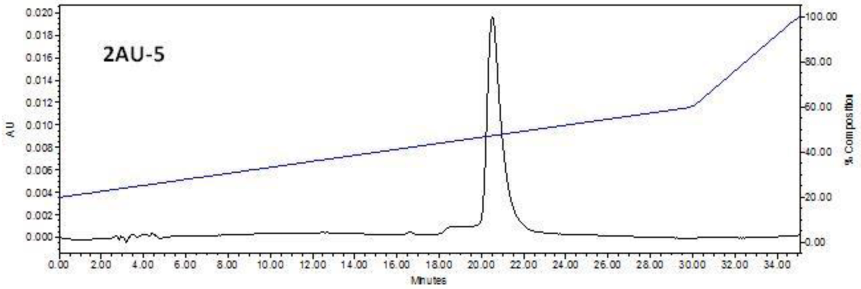
2AU-5
AU
% Composition
Minutes

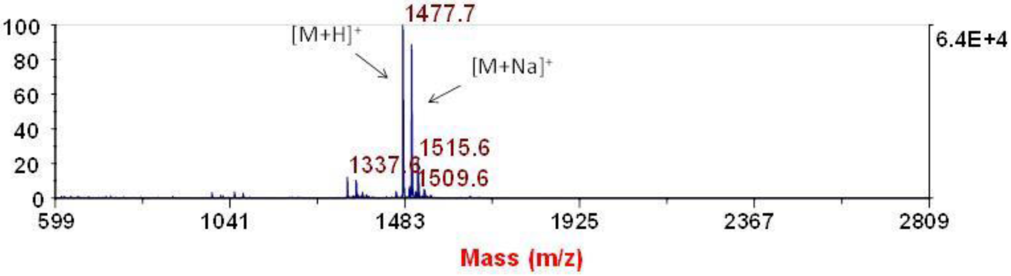
1477.7
[M+H]+
[M+Na]+
1515.6
1337.6
1509.6
6.4E+4
Mass (m/z)

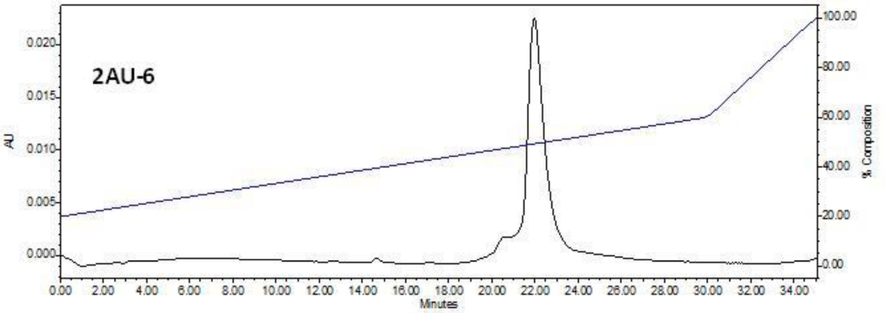
2AU-6
AU
% Composition
Minutes

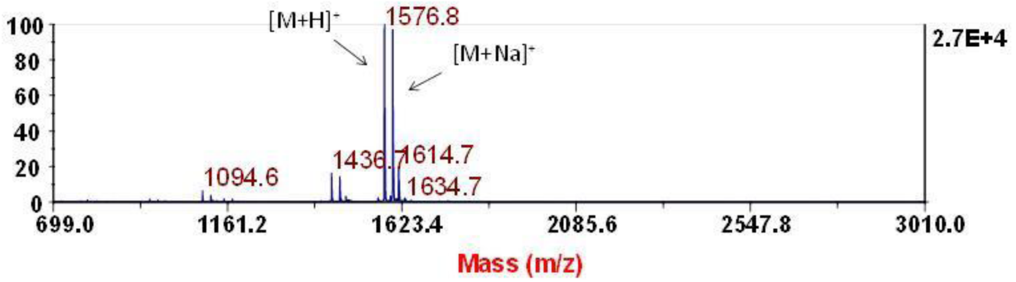
[M+H]+
1576.8
[M+Na]+
1094.6
1436.7
1614.7
1634.7
2.7E+4
Mass (m/z)

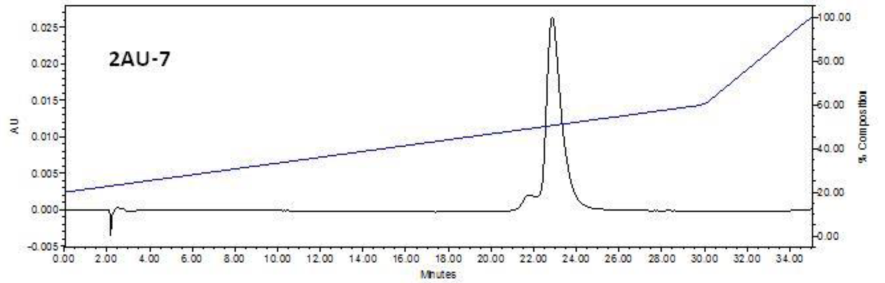
2AU-7
AU
% Composition
Minutes

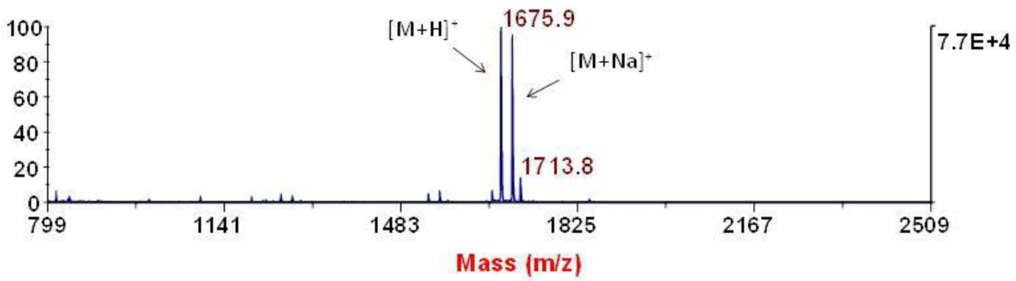
[M+H]+
1675.9
[M+Na]+
1713.8
7.7E+4
Mass (m/z)

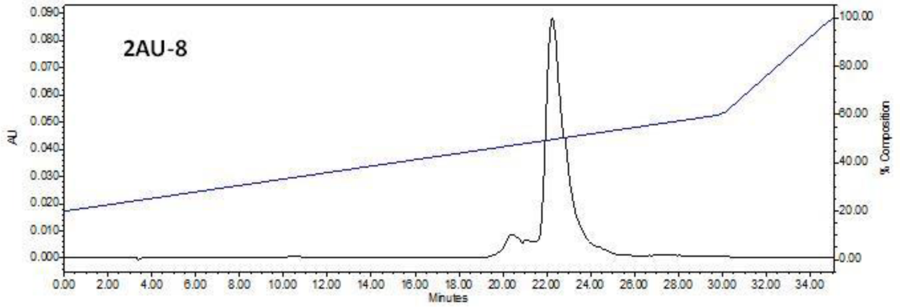

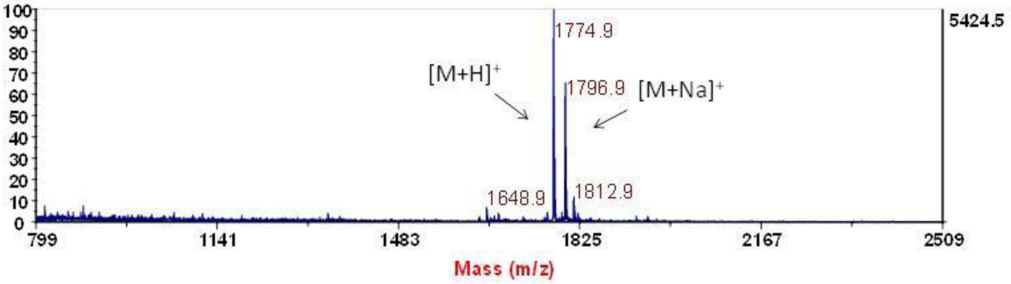

Supplementary Figure 22. HPLC chromatograms (blue: linear gradient of MeOH) and MALDI-TOF MS spectra of compounds.

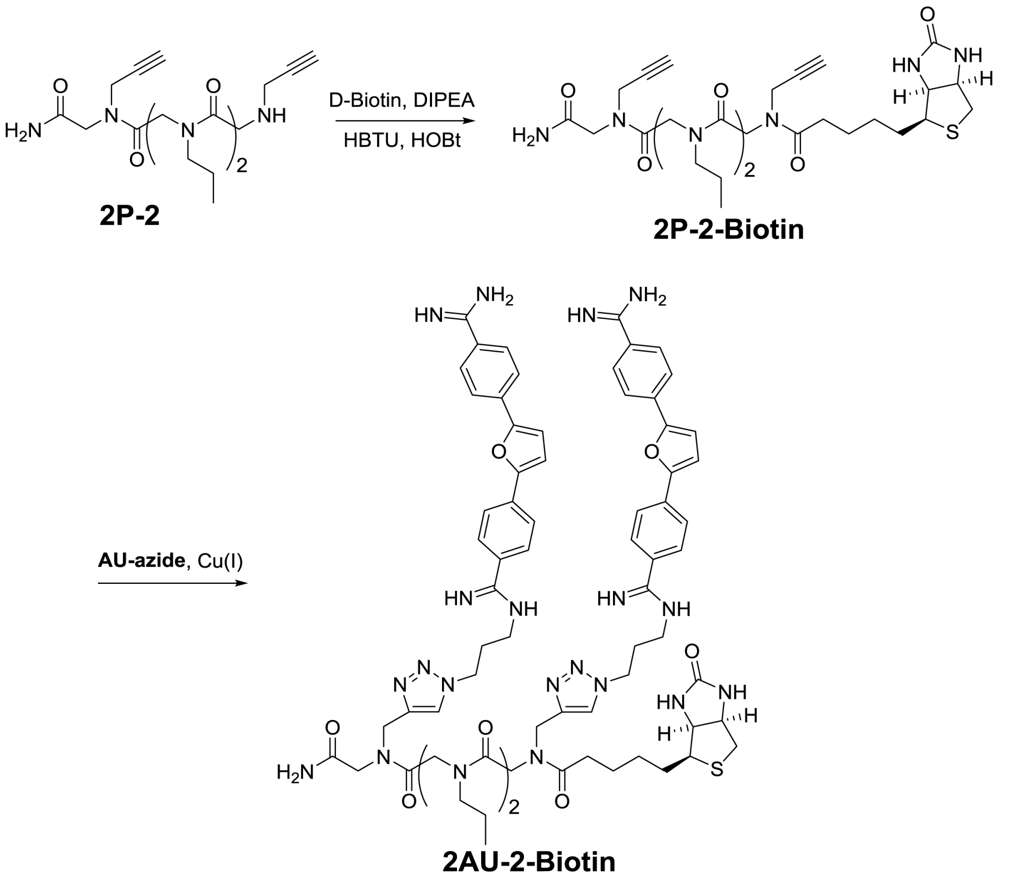

Supplementary Figure 23. Synthesis of **2AU-2-Biotin**.

Synthesis of 2AU-2-Biotin

2P-2-Biotin. **2P-2** on rink amide resin was shaken with a solution of D-biotin (5 eq.), HBTU (5 eq.) and HOBt (5 eq.) in DMSO at 37 °C for 1 h. **2P-2-Biotin** was cleaved from the resin by treatment with 60:40:1 of TFA:CH_2Cl_2 H_2O for 1 h and purified by HPLC.

2AU-2-Biotin. **2P-2-Biotin** (10 μmoles) was dissolved in DMF and Cu(I) catalyst (6 μmoles), DIPEA (10 μL) and **AU-azide** (20 μmoles) were added to the solution. The mixture was stirred overnight and **2AU-2-Biotin** (yield: 22%) was purified by using reverse phase HPLC as described above.

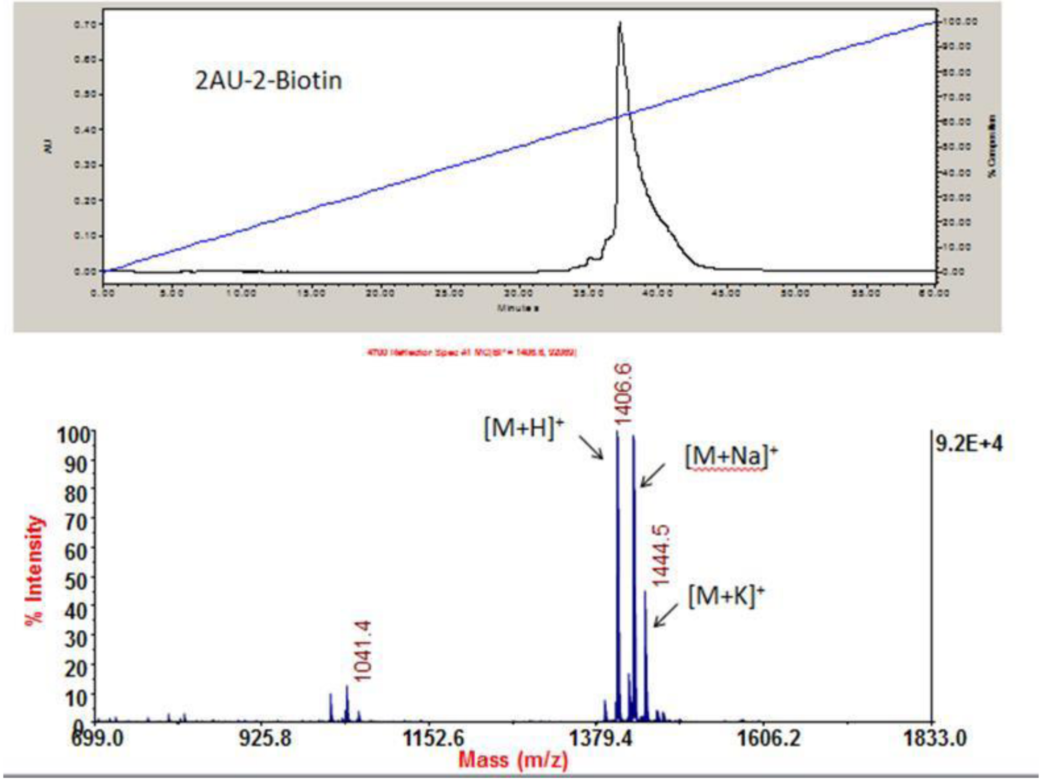

Supplementary Figure 24. HPLC chromatogram (blue: linear gradient of MeOH) and MALDI-TOF MS spectrum of **2AU-2-Biotin**.

Supplementary Tables

Supplementary Table 1. Substructures contained in the 104 compounds screened for binding to RNA base pairs.

	Structure	Count		Structure	Count		Structure	Count
1	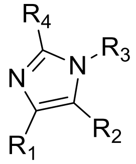	57	18	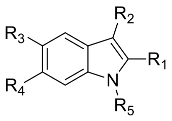	7	35	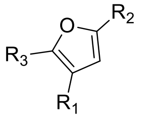	3
2	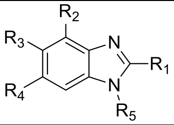	45	19	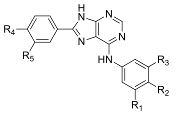	6	36	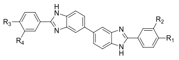	2
3	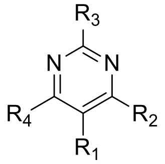	37	20	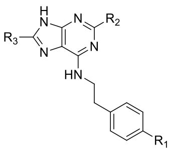	6	37	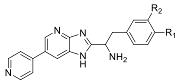	2
4	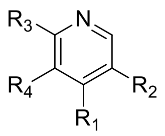	34	21	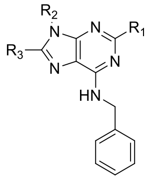	6	38	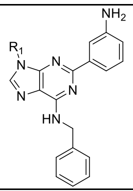	2
5	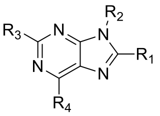	33	22	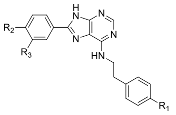	5	39	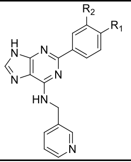	2
6	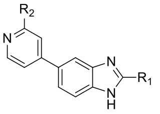	25	23	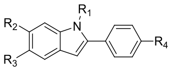	5	40	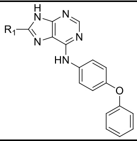	2
7	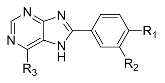	22	24	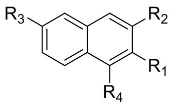	5	41	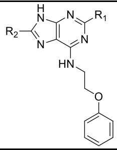	2
8	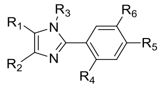	19	25	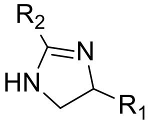	4	42	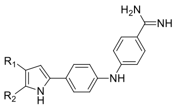	2
9	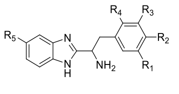	18	26	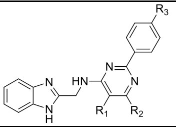	4	43	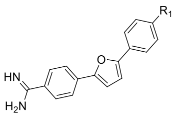	2
10	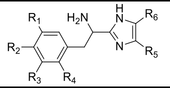	17	27	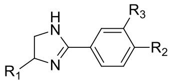	3	44	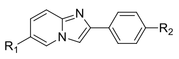	2
11	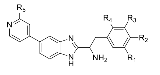	15	28	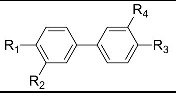	3	45	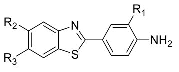	2

12	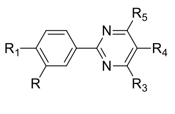	13	29	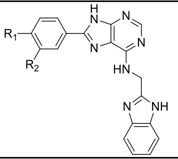	3	46	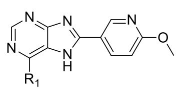	2
13	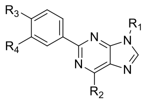	9	30	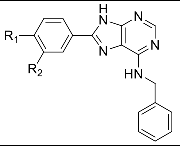	3	47	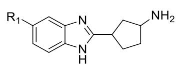	2
14	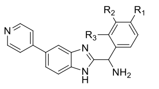	8	31	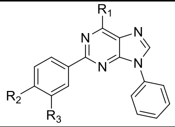	3	48	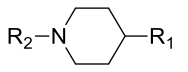	2
15	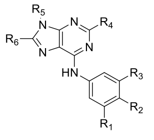	8	32	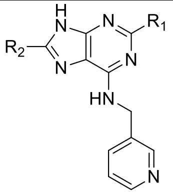	3	49	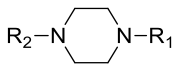	2
16	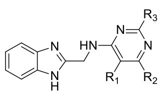	7	33	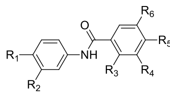	3	50	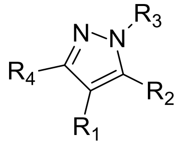	2
17	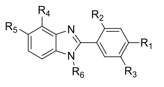	7	34	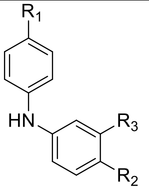	3			

Supplementary Table 2. Structures of hit compounds that bind RNA base pairs (>20% change in fluorescence intensity in the presence and absence of RNA). Asterisk (*) indicates selective binders.

Structure	Binding RNA(s)	Structure	Binding RNA(s)
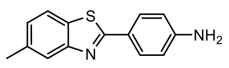	AAUU/GGCC	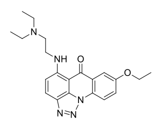	AUAU/GGCC/ GCGC
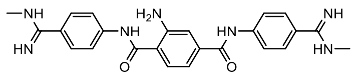 *	AAUU/AUAU	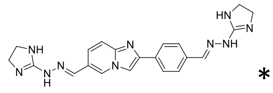 *	AAUU/AUAU
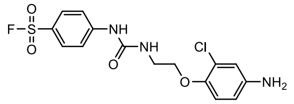	AAUU/GGCC/ GCGC	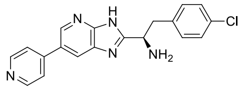	AAUU
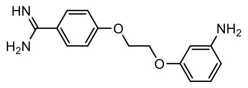	AAUU/AUAU/ GGCC/GCGC	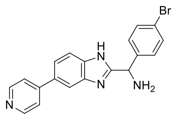 *	GCGC
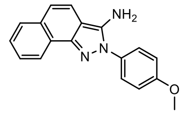	AAUU/AUAU/ GGCC	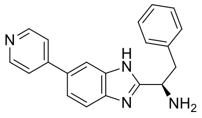	AAUU/AUAU
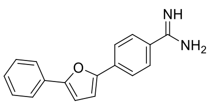 *[a]	AAUU/AUAU	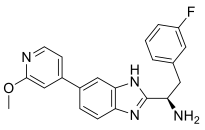 *	AAUU
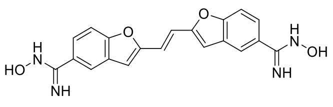	GCGC	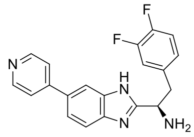	AUAU
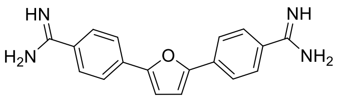 *	AAUU/AUAU	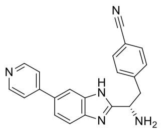	AAUU/GGCC/ GCGC
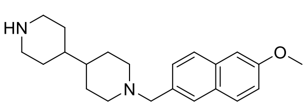	AUAU	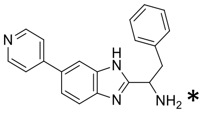 *	AAUU/AUAU

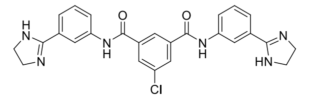	AAUU/AUAU/ GGCC/GCGC	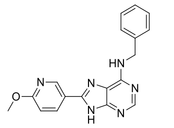	GCGC
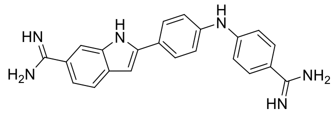	AAUU/AUAU/ GGCC/GCGC	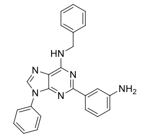	AUAU/GCGC
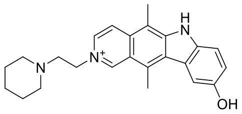	AUAU	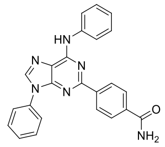	AUAU/GGCC/ GCGC
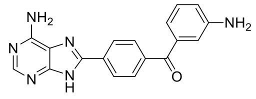	GCGC	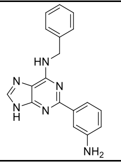	GGCC/GCGC
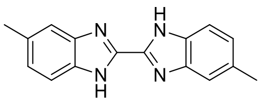	GGCC/GCGC	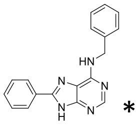 *	GGCC/GCGC
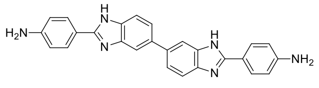	AAUU/AUAU/ GGCC/GCGC	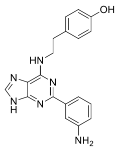	AAUU/GCGC

[a] This compound was selected from the group of compounds with <20 % of fluorescence change.

Supplementary Table 3. Sequence of primers for qPCR

RNA	Forward	Reverse	RNA	Forward	Reverse
18S rRNA	GTAACCCGTTGAACCCCATT	CCATCCAATCGGTAGTAGCG	FLNA	AGGGGACGGCCCTTTAAT	GTCGCTCTCAGGAACAGCAG
28S rRNA	AGAGGTAAACGGGTGGGGTC	GGGGTCGGGAGGAACGG	RPS6	CAAGAGAATGAAGGAGGCTAAGG	AAGCTCGCAGAGAGGAAAGTCT
5.8S rRNA	ACTCGGCTCGTGCGTC	GCGACGCTCAGACAGG	Rpl11	ACTTCGCATCCGCAAACTCT	TGTGAGCTGCTCCAACACCTT
45S rRNA	GAACGGTGGTGTGTCGTT	GCGTCTCGTCTCGTCTCACT	ENO1	GCCTCCTGCTCAAAGTCAAC	AACGATGAGACACCATGACG
5S rRNA	GGCCATACCACCCTGAACGC	CAGCACCCGGTATTCCCAGG	COX2	GCAGGGTTGCTGGTGGTAG	ATTTCATCTGCCTGCTCTGG
7SK	CCCCTGCTAGAACCTCCAAAC	CACATGCAGCGCCTCATTT	GNB2L1	TGGCTAACTGCAAGCTGAAGAC	ATCTGGAGAGACAGTCACCGTG
7SL	ATCGGGTGTCCGCACTAAGTT	CAGCACGGGAGTTTTGACCT	PKM2	TACCATGCGGAGACCATCAA	AGCAACGGGCCGGTAGAG
ACA16	GGCCCTTATCGAAGCTGCA	CGGCGACCGTCAAGGA	CLU	CCAGTGGAAGATGCTCAAC	CGAGTCAGAAGTGTGGGAAGC
ACA44	GTTTCCAAGGGCTGTGGCT	TGTACTGACCTGCGCTGTCAA	CANX	GCAACCACTTCCCTTCCAT	TCCGCCTCTCTCTTTACTGC
ACA61	CCTTTCCCATCGGATCTGAA	CCACATGCCATATACCAGATTACAAC	RPS9	AGACCCAGGTCTTCAAGCTG	ATGAAGGACGGGATGTTCAC
BC200	TGGCTCACGCCTGTAATCC	CCCAGGCAGGTCTCGAACT	GNAS	CAGTGGAGATGGGCGTCACTA	CGGCGGATGTTCTCAGTGT
HBI-36	CAGCACTGCCAAGTGACCC	ATATGTACCCAGCTGCATGCAG	COX1	CGATGCATACACCACATGAA	AGCGAAGGCTTCTCAAATCA
HBII-85	TGGATCGATGATGAGTCC	TGGACCTCAGTTCCGATGAGA	B2M	TGCTGTCTCCATGTTTGATGTATCT	TCTCTGCTCCCCACCTCTAAGT
HBII-420	ACTGGTCCAGGATGAAACCTAATT	CCTAGGAGCTGGTCTCAGTCCC	RPS3	TCCTCGGAGTTTCCCAGAC	TCCTAGGAGGGCTTGCTGT
U1	CCATGATCACGAAGGTGGTTT	ATGCAGTCGAGTTTCCCACAT	EEF1A1	TGCGGTTTTTGTCATCAAA	AAGAGTGGGGTGGCAGGTATTAG
U2	TTCTCGGCCTTTTGGCTAAG	CTCCCTGCTCCAAAAATCCA	Rpl27	TCCAAGGGGATATCCACAGA	CATGGGCAAGAAGAAGATCG
U4	GCCAATGAGGTTTATCCGAGG	TCAAAAATTGCCAATGCCG	RPL37	AAAACCAGAACATTTATTGCATGA	TCCGTGAAGGAACAACACCT
U5	TGGTTTCTCTTCAGATCGCATAAA	CCAAGGCAAGGCTCAAAAAAT	RPL3	CCTCCGTTCACCTGGATCT	CCAAGTCATCCGTGTCATTG
U6	CTCGCTTCGGCAGCACA	AACGCTTCACGAATTTGCGT	EEF2	GCACGTTCGACTCTTCACTG	CTGGAGATCTGCCTGAAGGA
U12	GCCCGAATCCTCACTGCTAA	TCGCAACTCCCAGGCATC	ND2	GCCCTAGAAATAAACATGCTA	GGGCTATTCCTAGTTTTATT
U87	ATGGGATCATGGAGCAGCTG	TCACACCCATGACTGCCACT	TPT1	CATGATTATCTACCGGGACCTCAT	AACCCGTCCGCGATCTC
U105	CCCCTATCTCTCATGATGAACACATAT	CCCCATCTCTTCTTCAGAGCG	TMSB4X	AGACCAGACTTCGCTCGTA	CTGCTTCGTTCTCCTGTT
tRNA(Lys)	CGGCTAGCTCAGTCGGTAGA	CCCACGACCCTGAGATTAAG	RPL4	GCTCTGGCCAGGGTGCTTTTG	ATGGCGTATCGTTTTTGGGTTGT
tRNA(Ala)	GGGGGTGTAGCTCAGTGGTA	AGGCCTCATACATGCAAAGC	CCT5	ACAGCCTTTGCTGCCATTAT	GCCCTTTCCTCATCATCAAG
tRNA(Cys)	GCTCAGGGGTAGAGCATTTG	ACCGGGGACTTCTGGATCT	PRDX1	GGGCACACAAAGGTGAAGTC	GCTGTTATGCCAGATGGTCAG
tRNA(His)	GCAGTGACTGTATAGTGGTTAGCA	GTGGCCACAACACAGAGTG	PFN1	GATCACCGAACATTTCTGGC	AAACGTTCGTCAACATCACG
tRNA(Ser)	TAGTCGTGGCCGAGTGGTTA	GGAAACCCCAATGGATTTCTA	Rps24	AAGACCACACCGGATGTCATC	TGCCAAAGCCAGTTGTCTTG
tRNA(Val)	TGGTTATCACGTTCGCCTAA	GTTTCGAACCGGGGACCT	PPIA	TTATTTGGGTTGCTCCCTTC	AAGTGTGCCAAATCTGCAAG
tRNA(Arg)	CAGTGGCGCAATGGATAAC	CAGGAGTCGAACCTGGAATC	LDHB	TGGCGTGTGCTATCAGCATT	GCTTATCTTCCAAAACATCCACAAG
tRNA(Gln)	TGGTTAGCACTCTGGACTCTGA	AGGTTCCACCGAGATTTGAA	LGALS1	AAGCTGCCAGATGGATACGAA	CGTCAGCTGCCATGTAGTTGA
tRNA(Ile)	CAGTTGGTTAGAGCGTGGTG	CCACGACCTTGGCGTTATTA	PGK1	ATGGATGAGGTGGTGAAAGC	CAGTGCTCACATGGCTGACT

tRNA(Thr)	TAGCTGGTTAAAGCGCCTGT	GAACCCAGGATCTCCTGTTTACT	BASP1	CAATGCCAATCCTCCATTCT	AACTACAGGTGCACCCAACC
tRNA(Asn)	CAATGGGTTAGCGCGTTC	AACCACCAACCTTTCGGTTA	YBX1	TGATGGAGGGTGCTGACAAC	CCTGCGGAATCGTGGTCTAT
tRNA(Glu)	CTGGTGGTCTAGTGGCTAGGA	CTGGCCGGGAATCGAAC	KRT7	TGGAGGACTTCAAGAATAAGTACGAA	TCATGTAGGCAGCATCCACATC
tRNA(Ini)	CATAACCCAGAGGTCGATGG	TAGCAGAGGATGGTTTCGAT	CYTB	AATTCTCCGATCCGTCCCTA	GGAGGATGGGGATTATTGCT
tRNA(Phe)	TCAGTTGGGAGAGCGTTACA	AGGGTTGAACCAGGGAACTT	NONO	CCCTTACAGTTCGAAACCTT	ATGACTACAGCCCTCTCTAC
tRNA(Trp)	GCGCGTCTGACTCCAGAT	ACGTGATTTGAACACGCAAC	ND1	ATACCCCCCATTCCGCTACGAC	GTTTGAGGGGGAATGCTGGAGA
tRNA(Asp)	TCTGCCTGTCATGTGGAGAC	CCTGTTGGGGACTCAAACTC	ALDOA	CGGGAAGAAGGAGAACCTG	GACCGCTCGGAGTGTACTTT
tRNA(Gly)	GCATTGGTGGTTCAGTGGTA	ATTGGCCGGGAATTGAAC	P4HB	GCAAACTGAGCAACTTCAAA	TTCTTCAGGCCAAAGAACTC
tRNA(Leu)	GGTCTAAGGCGCTGGATTAAG	CCCCCGAAGAGACTGGAG	LDHA	ACCCAGTTTCCACCATGATT	CCCAAAATGCAAGGAACACT
tRNA(Pro)	GGGGTATGATTCTCGCTTAGG	ATTTGAACCCGGGACCTCT	APP	GCTGGCTGAACCCCAGATT	CCCACTTCCCATTCTGGACAT
tRNA(Tyr)	CTGGTAGAGCGGAGGACTGT	GGAATTGAACCAGCGACCTA	FOLR1	GCACCACAAGGAAAAGCCAG	CATTCTTCCTCCAGGGTCGAC
tRNA(Sec)	GGCTTCAAACCTGTAGCTGTC	CCGAAATGGAATTGAACCAC	ACTB	CTGGAACGGTGAAGGTGACA	AAGGGACTTCCTGTAACAATGCA
FTH1	GGCAAAGTTCTTCAAAGCCA	CATCAACCGCCAGATCAAC	RPL13	CCCGTCCGGAACGTCTATAA	CTAGCGAAGGCTTTGAAATTCTTC
GAPDH	TGCACCACCAACTGCTTAGC	GGCATGGACTGTGGTCATGAG	RPS21	GGCGAGTTCGTGGACCTGTA	GGATGGATGCGTGGTCCTT
ATP6	TTCTGGAATGACTCCTTTGC	TTGGCCAGAATGAACTTGAA	S100A6	CTGCAGGATGCTGAAATTGC	GGAAGTTCACCTCCTGGTCCTT
RPL8	AGATGGGTTTGTCAATTCGG	CAAGAAGACCCGTGTGAAGC			

Supplementary Table 4. Characterization of modularly assembled molecules including HPLC retention times, and calculated and observed masses.

Compound	Molecular Formula	t_R (min)[a]	Mass (Calculated)	MALDI-TOF (+)-Mass (Found)
**2AU-0**	$C_{52}H_{55}N_{17}O_4$	14.6	981.5 (M)	982.6 (M+H)
**2AU-1**	$C_{57}H_{64}N_{18}O_5$	16.6	1080.5 (M)	1081.4 (M+H)
**2AU-2**	$C_{62}H_{73}N_{19}O_6$	17.7	1179.6 (M)	1180.5 (M+H)
**2AU-3**	$C_{67}H_{82}N_{20}O_7$	18.8	1278.7 (M)	1279.8 (M+H)
**2AU-4**	$C_{72}H_{91}N_{21}O_8$	19.2	1377.7 (M)	1378.7 (M+H)
**2AU-5**	$C_{77}H_{100}N_{22}O_9$	20.5	1476.8 (M)	1477.7 (M+H)
**2AU-6**	$C_{82}H_{109}N_{23}O_{10}$	22.0	1575.9 (M)	1576.8 (M+H)
**2AU-7**	$C_{87}H_{118}N_{24}O_{11}$	22.9	1674.9 (M)	1675.9 (M+H)
**2AU-8**	$C_{92}H_{127}N_{25}O_{12}$	22.3	1774.0 (M)	1774.8 (M+H)
**2AU-2-Biotin**	$C_{72}H_{87}N_{21}O_8S$	38.0[b]	1405.7 (M)	1406.6 (M+H)

[a] gradient of 20-60% MeOH in H_2O with 0.1% TFA over 30 min
[b] gradient of 0-100% MeOH in H_2O with 0.1% TFA over 60 min

Supplementary References

1. Fu HY, Doucet H. Methyl 2-Furoate: An Alternative Reagent to Furan for Palladium-Catalysed Direct Arylation. *Eur. J. Org. Chem.* **2011**, 7163-7173 (2011).

2. Das BP, Boykin DW. Synthesis and antiprotozoal activity of 2,5-bis(4-guanylphenyl)furans. *J. Med. Chem.* **20**, 531-536 (1977).

3. Pushechnikov A*, et al.* Rational design of ligands targeting triplet repeating transcripts that cause RNA dominant disease: application to myotonic muscular dystrophy type 1 and spinocerebellar ataxia type 3. *J. Am. Chem. Soc.* **131**, 9767-9779 (2009).
